# Massive Bioaccumulation and Self‐Assembly of Phenazine Compounds in Live Cells

**DOI:** 10.1002/advs.201500025

**Published:** 2015-06-05

**Authors:** Kyoung Ah Min, Walajapet G. Rajeswaran, Rudolf Oldenbourg, Grant Harris, Rahul K. Keswani, Mason Chiang, Phillip Rzeczycki, Arjang Talattof, Mahwish Hafeez, Richard W. Horobin, Scott D. Larsen, Kathleen A. Stringer, Gus R. Rosania

**Affiliations:** ^1^ Department of Pharmaceutical SciencesUniversity of Michigan College of Pharmacy428 Church StAnn ArborMI48109USA; ^2^ Department of Medicinal ChemistryUniversity of Michigan College of Pharmacy428 Church StAnn ArborMI48109USA; ^3^ Marine Biological LaboratoriesWoods HoleMA02543USA; ^4^ School of Life SciencesThe University of GlasgowUniversity AvenueGlasgowG12 8QQScotlandUK; ^5^ Department of Clinical, Social and Administrative SciencesUniversity of Michigan College of PharmacyAnn ArborMI48109USA

**Keywords:** aggregation, biocrystals, chromophores, clofazimine, medicinal chemistry

## Abstract

Clofazimine is an orally administered drug that massively bioaccumulates in macrophages, forming membrane‐bound intracellular structures possessing nanoscale supramolecular features. Here, a library of phenazine compounds derived from clofazimine is synthesized and tested for ability to accumulate and form ordered molecular aggregates inside cells. Regardless of chemical structure or physicochemical properties, bioaccumulation is consistently greater in macrophages than in epithelial cells. Microscopically, some self‐assembled structures exhibit a pronounced, diattenuation anisotropy signal, evident by the differential absorption of linearly polarized light, at the peak absorbance wavelength of the phenazine core. The measured anisotropy is well above the background anisotropy of endogenous cellular components, reflecting the self‐assembly of condensed, insoluble complexes of ordered phenazine molecules. Chemical variations introduced at the R‐imino position of the phenazine core lead to idiosyncratic effects on the compounds' bioaccumulation behavior as well as on the morphology and organization of the resulting intracellular structures. Beyond clofazimine, these results demonstrate how the self‐assembly of membrane permeant, orally bioavailable small molecule building blocks can endow cells with unnatural structural elements possessing chemical, physical, and functional characteristics unlike those of other natural cellular components.

## 1 Introduction

In mammals, macrophages orchestrate many key physiological functions, including wound healing and regeneration, clearance of apoptotic and necrotic cells, recognition of foreign antigens, defense against invading pathogens, and mounting of protective immune responses.^1^ In addition, macrophages play a physiological role in the disposition of lipophilic, poorly soluble small molecule chemical agents. In the liver, for example, Kupffer cells sequester lipids, cholesterol, fat soluble vitamins, xenobiotics, and drugs.^2^ Perhaps it is not too surprising that clofazimine, an old but highly effective antibiotic that is included in the World Health Organization's list of essential medications and part of the standard treatment of leprosy,^3^ has been found to massively bioaccumulate in macrophages.^4^ In these cells, clofazimine forms crystal‐like drug inclusions (CLDIs): highly organized, insoluble molecular complexes that are predominantly found in membrane‐bound compartments within the cytoplasm.^4^ Although drug crystal formation has generally been regarded as an unwanted side effect, clofazimine is a well‐tolerated, clinically useful drug. Thus, in the case of clofazimine, CLDIs may function as a biocompatible, intracellular drug depot mechanism. More generally, CLDI formation could be exploited as a means to target drugs to macrophages in living organisms and to endow these cells with unnatural structural and functional elements for diagnostic or therapeutic purposes.

Related to clofazimine, Neutral Red is a phenazine compound that undergoes accumulation in lysosomes,^5^ while Janus Green B is another phenazine compound that undergoes electrical potential dependent accumulation in mitochondria.^6^ However, neither of these compounds exhibits the massive intracellular bioaccumulation or intracellular self‐assembly properties that are characteristic of clofazimine. Furthermore, because of differences in membrane partitioning, the transport mechanisms mediating the cellular uptake and intracellular distribution of clofazimine may be different from that of less lipophilic, more soluble phenazine derivatives.^8^ Therefore, to explore whether other phenazine compounds could provide a good a starting point for developing new kinds of self‐assembling intracellular elements for drug delivery and bioimaging applications, we decided to examine the impact of variations in lipophilicity and chemical structure on the intracellular uptake and trafficking of phenazine compounds. For this purpose, we synthesized a small, focused library of phenazine derivatives of clofazimine, and assayed their bioaccumulation and self‐assembly properties in RAW264.7 macrophages and Madin‐Darby Canine Kidney (MDCK) epithelial cells.

Because phenazines are chromophores with a broad absorbance peak in the range of visible wavelengths, the interaction of the phenazine ring with monochromic, polarized light could be useful to specifically probe the intracellular accumulation, distribution, and molecular organization of the phenazine compounds. Accordingly, a quantitative polarization microscope (LC‐PolScope)^9^ was adapted to assay the formation of condensed, ordered molecular aggregates of the compounds in live cells. With this instrument, we measured the manner in which the intracellular inclusions differentially influenced the transmittance of polarized light—an optical property known as diattenuation anisotropy.^10^ By relating the extent of bioaccumulation to the measured optical properties of the intracellular inclusions formed by different phenazine derivatives, we determined that certain chemical modifications at the R‐imino group promoted the self‐assembly of phenazines, specifically in macrophages. The influence of these variations on bioaccumulation and structure formation seemed highly idiosyncratic. However, all compounds tended to preferentially accumulate in macrophages relative to epithelial cells, regardless of their chemical structure or physicochemical properties.

## 2 Results and Discussion

**Scheme 1:**
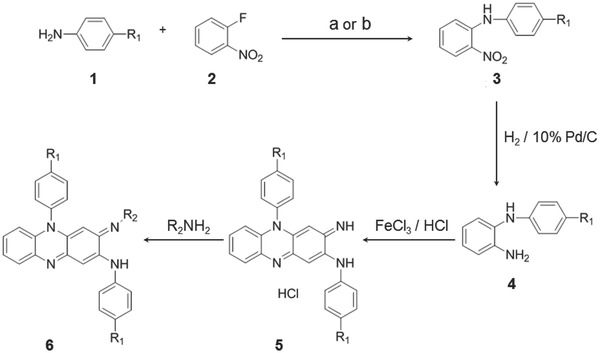
Synthesis of phenazine derivatives. Treatment of aniline derivatives **1** with 2‐fluoronitrobenzene **2** (using reagents: a) KF/K_2_CO_3_ or b) KOH/DMSO) gave the secondary amine derivatives **3** in 22–63% yield. Reduction of the nitro group was carried out using 10% Pd/C catalyst under hydrogen atmosphere to yield the diamine **4** in 55–99% yield. Then the diamine **4** was oxidized in aqueous ferric chloride solution to give the corresponding phenazine salts **5** in 70–96% yield. The phenazine salts **5** on treatment with variety of primary amines gave the corresponding phenazine derivatives **6** in 10–85% yield. These methods are elaborated in detail in the Supporting Information.

**Figure 1:**
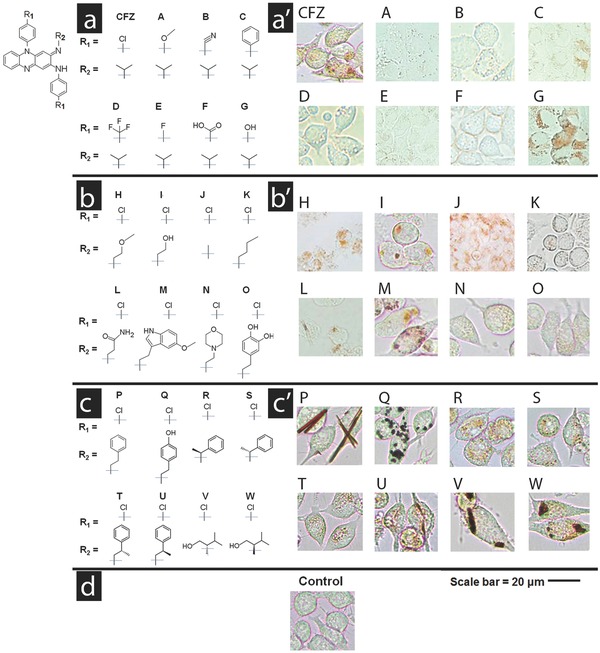
Three series of phenazine derivatives of clofazimine were synthesized to probe the relationship between clofazimine's chemical structure a–c) and its cellular staining patterns a′–c′). The common phenazine core shared by all phenazine compounds is shown in the top left corner of the figure. In the first series of derivatives a,a′), the chlorophenyl groups of clofazimine were substituted with different functionalities. In the second series b,b′), the isopropyl R‐imino group of clofazimine was substituted with achiral functional groups. In the third series c,c′), the isopropyl R‐imino group of clofazimine was substituted with additional functional groups that probed the effects of an added stereochemical center. Scale bar = 20 μm.

First, we synthesized a focused library of clofazimine derivatives (**Scheme** **1**), by replacing the chlorophenyl moieties of clofazimine with different aromatic substituents (**Figure** **1**a and Table S1 (Supporting Information), Compounds A–G). When these compounds were incubated with cells, they exhibited similar or decreased intracellular staining relative to clofazimine, irrespective of their higher or lower lipophilicity relative to clofazimine (Figure 1a' and Table S2, Supporting Information). Small changes in the chemical structure of clofazimine were associated with visibly different cellular staining pattern in macrophages. In this first series of phenazine compounds (Compounds A–G), the chlorophenyl moiety of clofazimine was replaced with various other closely related substituents, yet most of these substituents (Compounds A–F) led to decreased cellular staining in relation to clofazimine. Only the replacement of chlorines with hydroxyls led to a staining pattern similar to that of clofazimine (Compound G).

For comparison, we synthesized a second, focused series of derivatives, in which the isopropyl group at the R‐imino position of clofazimine was replaced with different achiral substitutents (Figure 1b and Table S3, Supporting Information, Compounds H–Q). Compared to clofazimine, replacing the isopropyl group with other functional groups at the R‐imino position generally led to similar or greater staining of macrophages (Figure 1b′ and Table S4, Supporting Information, Compounds H–Q). Three derivatives yielded cellular staining patterns comparable in morphology and intensity to those of clofazimine (Compounds H, I, and M) while two derivatives yielded more prominent staining patterns (Compounds P and Q). The most intense staining was associated with the formation of condensed cytoplasmic inclusions, either amorphous or crystal‐like in morphology (Figure 1b′ and Table S4, Supporting Information; Compounds P and Q).

**Figure 2:**
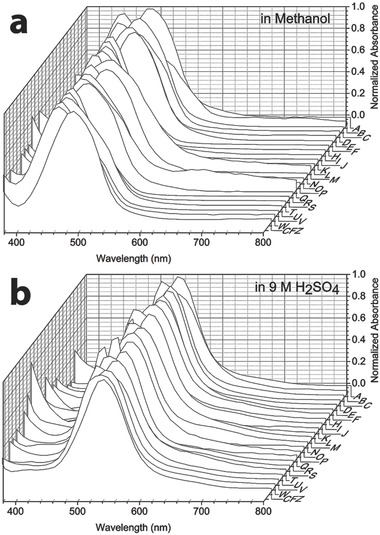
Absorbance spectra of phenazine compounds were very similar to that of clofazimine. Absorption peaks were between 450 and 490 nm when measured in methanol and between 510 and 540 nm when measured in H_2_SO_4_.

To assess the extent to which specific interactions with chiral components present in cells (or the cell culture medium) affected the bioaccumulation and self‐organization behavior of the compounds, we proceeded to synthesize and screen a third, focused series of phenazine derivatives, which incorporated a stereochemical center at the R‐imino position (Figure 1c and Table S5, Supporting Information, Compounds R–W). As observed in the second series of achiral R‐imino phenazines, small variations in the chemical structure and physicochemical properties of these chiral R‐imino phenazine compounds led to pronounced differences in cellular staining (Figure 1c′ and Table S6, Supporting Information). Three of the six chiral R‐imino derivatives exhibited prominent cellular staining, associated with the formation of yellow, orange, red, or brown cytoplasmic inclusions (Figure 1c′ and Table S6, Supporting Information, Compounds U, V, and W). In control experiments, untreated cells showed no visible yellow or brown staining when viewed using the same optical set up under transmitted, brightfield illumination (Figure 1d, Control). Furthermore, staining was also not observed when cells were fixed prior to incubation with the compounds (Table S6, Supporting Information), indicating that the observed staining patterns reflected underlying differences in physiological, small molecule transport mechanisms present in live cells. Because all phenazine compounds exhibited similar absorbance spectra under different conditions (**Figure** **2**a,b), the variations in cellular staining patterns most likely reflected differences in the accumulation and distribution of the compounds in the cells, independently from the compounds' optical properties.

**Figure 3:**
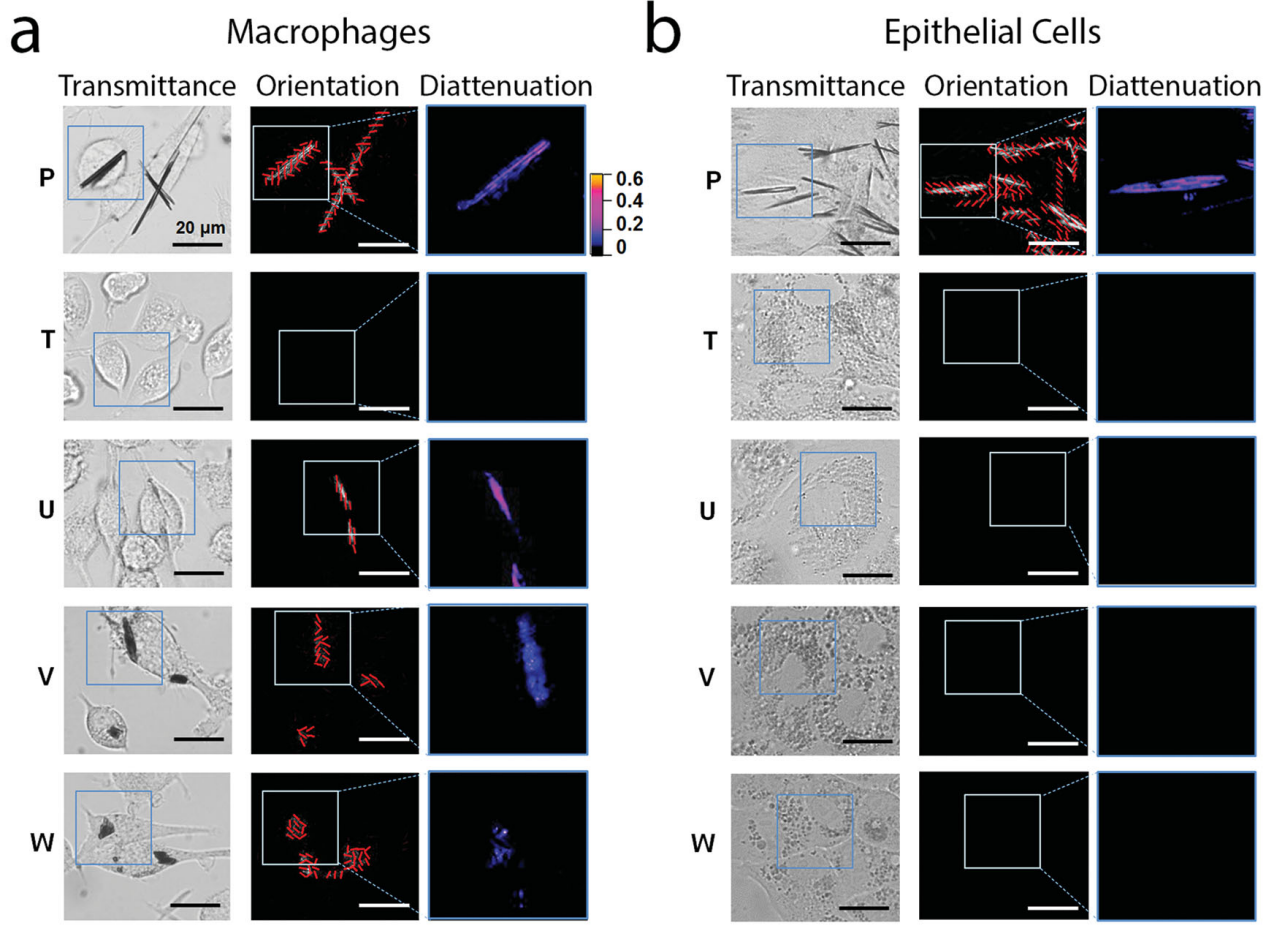
Quantitative polarization microscopy of macrophages and epithelial cells incubated with the different phenazine compounds revealed cell type‐specific differences in transmittance, diattenuation anisotropy, and the orientation of the polarization axis maximal transmittance of the intracellular inclusions. For the experiments, live RAW264.7 macrophages a) or MDCK epithelial cells b) were incubated for 72 h with clofazimine or other phenazine analogs and analyzed with the diattenuation anisotropy microscope imaging set up, using monochromatic light of 546 nm wavelength. Transmittance corresponds to the image map of the transmitted light intensity at 546 nm wavelength (white corresponds to 100% transmittance and black corresponds to 0% transmittance) and orientation corresponds to the measured direction of maximal light transmittance of linearly polarized light across the sample, indicated with a grid of red lines superimposed on the image. Diattenuation corresponds to the quantitative diattenuation anisotropy image map measured using linearly polarized light of 546 nm wavelength. The color‐gradient calibration bar corresponds to diattenuation anisotropy values ranging from 0 to 0.6. Scale bar = 20 μm.

Next, we proceeded to characterize the optical properties of the inclusions using a quantitative, polarization microscope^9^ to perform diattenuation anisotropy measurements on cells incubated with the different compounds.^10^ By transmitting linearly polarized, monochromatic light of wavelengths near the absorbance maxima of the phenazine molecules, the diattenuation anisotropy signal can be used to detect and quantify the presence of ordered aggregates of phenazine molecules in live cells. For isotropic, disordered intracellular inclusions, polarized light is expected to be transmitted in the same manner irrespective of the orientation of the polarization vector, resulting in diattenuation anisotropy close to 0. For ordered intracellular inclusions of phenazine molecules (as occurs when molecules are aligned with each other), the diattenuation anisotropy should increase, as the ordered molecular aggregates will preferentially transmit light that is polarized in a particular direction. Accordingly, by comparing the diattenuation anisotropy image maps for treated and untreated control cells, we observed clear diattenuation anisotropy signals in association with dense inclusions formed specifically by phenazine compounds (see Compounds P, U, V, and W in **Figure** **3**). Interestingly, Compounds U, V, and W exhibited optically anisotropic inclusions only in macrophages, whereas Compound P formed optically anisotropic inclusions in both macrophages (Figure 3a) and epithelial cells (Figure 3b). By visual inspection, we observed significant variations in the molecular organization of the inclusions formed by different compounds: Some compounds formed monolithic aggregates comprised of a single domain with uniform orientation (Compound P; Figure 3a,b), while other compounds formed complex aggregates comprised of segregated domains with subdomains organized in different directions (Compounds U, V, and W; Figure 3a).

**Figure 4:**
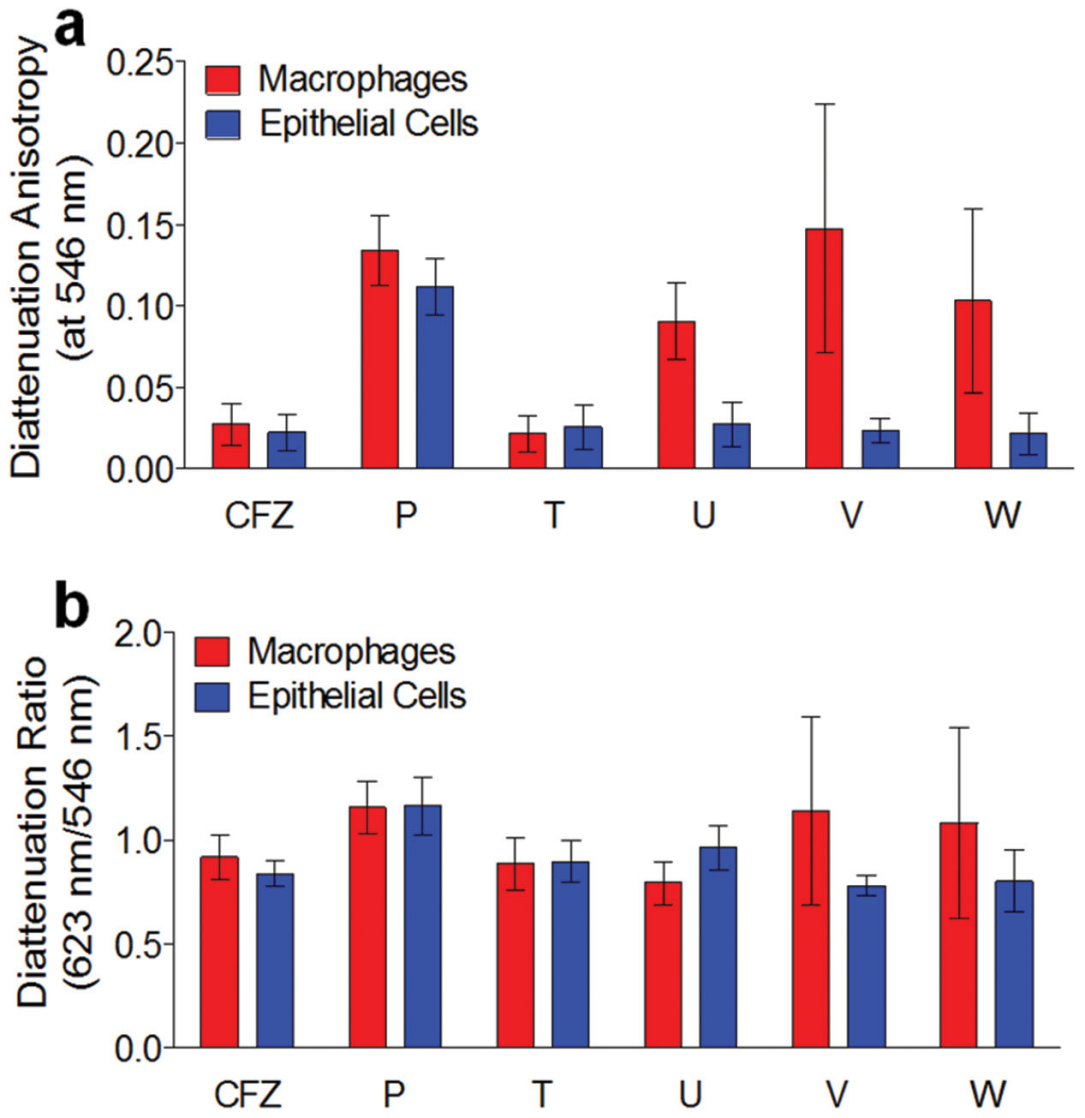
Quantitative comparison of the diattenuation anisotropy measurements of intracellular inclusions formed by clofazimine and other phenazine derivatives showing the most prominent diattenuation anisotropy signals (Compounds P, U, V, and W). Compound T was included as well, since it corresponds to the mirror image (chiral pair) of Compound U. a) Compared to clofazimine, Compounds U, V, and W show stronger diattenuation anisotropy signals in macrophages, while Compound P shows stronger diattenuation anisotropy signals in both macrophages and epithelial cells. b) The ratio of 623 nm/546 nm diattenuation anisotropy signals of the phenazine compounds was similar to that of clofazimine. Suggesting greater variability in the organization of the intracellular inclusions, Compounds V and W exhibited greater standard deviations, corresponding to greater differences in the measured diattenuation anisotropy ratios amongst individual inclusions.

We quantitatively confirmed that Compounds P and Q showed higher diattenuation anisotropy in both macrophages and epithelial cells than seen with clofazimine (**Figure** **4**a). Notably, chiral Compounds U, V, and W exhibited even higher anisotropy as compared to clofazimine but only in macrophages (Figure 4a). The measured diattenuation anisotropy signals for all chiral compounds were well above the background, diattenuation anisotropy signal of untreated cells (Figure 4a). On average, the anisotropy diattenuation ratio measured at a wavelength of 546 nm relative to 623 nm did not reveal significant wavelength‐dependent differences in diattenuation anisotropy (Figure 4b), although some compounds exhibited higher variability in the measured diattenuation anisotropy ratios in macrophages than in epithelial cells (Figure 4; Compounds V and W).

**Figure 5:**
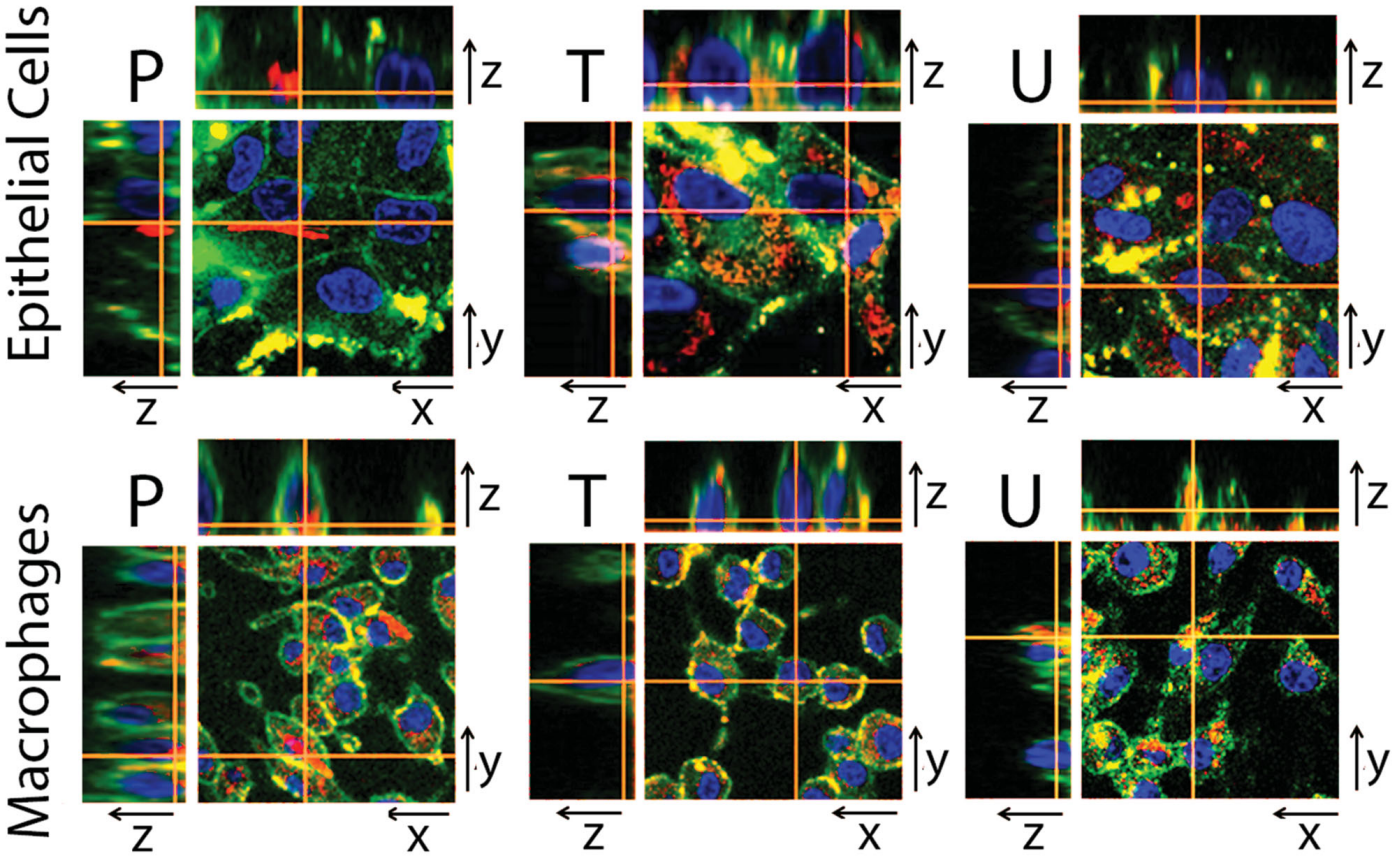
Confocal fluorescence microscopy of RAW264.7 macrophages or MDCK epithelial cells after incubation with fluorescent phenazine Compounds, P, T, or U. 2D images in xy planes show the location of cell nuclei (blue) and plasma membranes (green), together with the corresponding, intracellular location of phenazine compounds (red). 3D reconstructions of optical sections through orthogonal planes (*xz* and *yz* planes) confirm intracellular, cytoplasmic signals of compounds (red), in relation to the position of the nuclei and plasma membrane signals. Scale bar = 20 μm.

In relation to the compounds' chemical structures, the self‐assembly and resulting optical properties of intracellular structures formed by phenazine compounds appeared to be highly idiosyncratic: only one pair of enantiomers (Figure 3 and 4a, Compounds T and U) exhibited enantioselective differences in their optical anisotropy signal and this was observed only in macrophages. Compunds V and W did not show enantioselective differences in diattenuation anisotropy (**Figure** **5**a,b). The other pair of enantiomers (Compounds R and S) did not yield a measurable, diattenuation anisotropy signal in either macrophages or epithelial cells. Furthermore, Compound P, an achiral phenazine compound, formed inclusions with strong diattenuation anisotropy signals in both macrophages and epithelial cells (Figure 3 and 4), indicating that the formation of ordered, R‐imino phenazine aggregates can occur regardless of stereochemistry.

To confirm the intracellular localization of the inclusions, we took advantage of the fluorescence properties of the phenazine Compounds P, T, and U. Following intracellular accumulation, these compounds exhibited strong fluorescence excitation and emission signals that were visible through the standard (tetramethylrhodamine isothiocyate) TRITC channel of a fluorescence, confocal microscope. Accordingly, we acquired confocal optical sections through cells incubated with these compounds to confirm that the inclusions were intracellularly localized (Figure 5). For counterstaining, cells were also incubated with an orthogonally fluorescent nuclear marker (Hoechst 33342), as well as an orthogonally fluorescent, plasma membrane‐specific marker (FM‐143). FM‐143 yielded a green, plasma membrane signal in the fluorescein isothiocyanate (FITC) channel of the microscope, while Hoechst 33342 yielded a blue, nuclear signal in the 4′,6‐diamidino‐2‐phenylindole (DAPI) channel. In both macrophages and epithelial cells, optical sections through the cells clearly revealed that Compounds P, T, and U (red signals) were localized at the periphery of the cell nuclei (blue signal) and within the confines of the cells' plasma membrane (green signal) (Figure 5).

**Figure 6:**
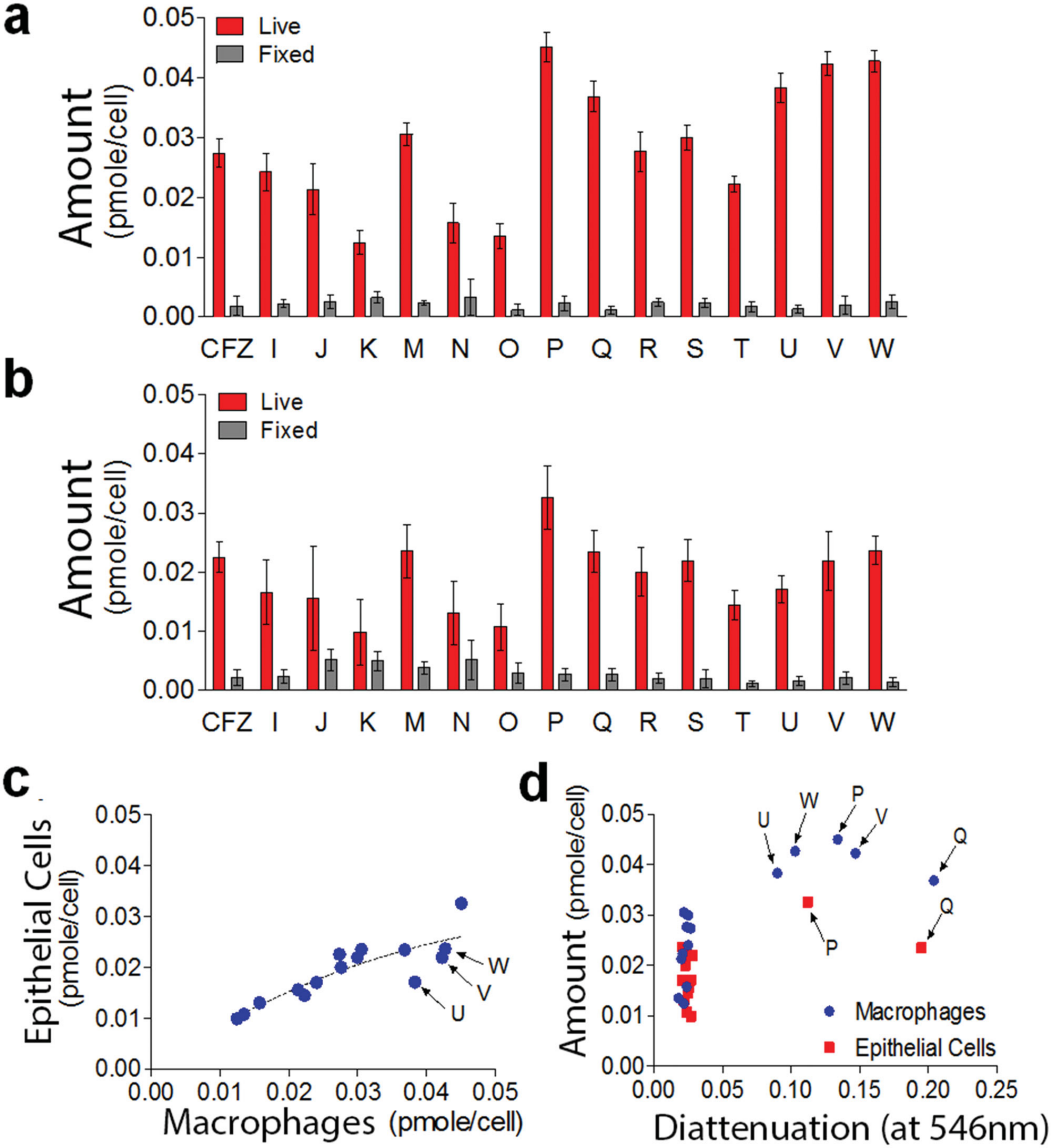
Measured differences in the accumulation of clofazimine or its phenazine derivatives (in pmols per cell) in RAW264.7 macrophages or MDCK epithelial cells following a 72 h incubation period. a) Measurements indicate significant differences in the accumulation of the compounds in live versus fixed macrophages. b) Measurements also indicate significant differences in the accumulation of the compounds in live versus fixed epithelial cells. c) Plot of mass accumulation of compounds in macrophages versus epithelial cells reveals overall trend towards higher accumulation of the compounds in macrophages. d) Plot of the cellular accumulation of phenazine compounds in macrophages and epithelial cells in relation to the diattenuation anisotropy of the resulting intracellular inclusions, measured using linearly polarized light at 546 nm wavelength.

**Figure 7:**
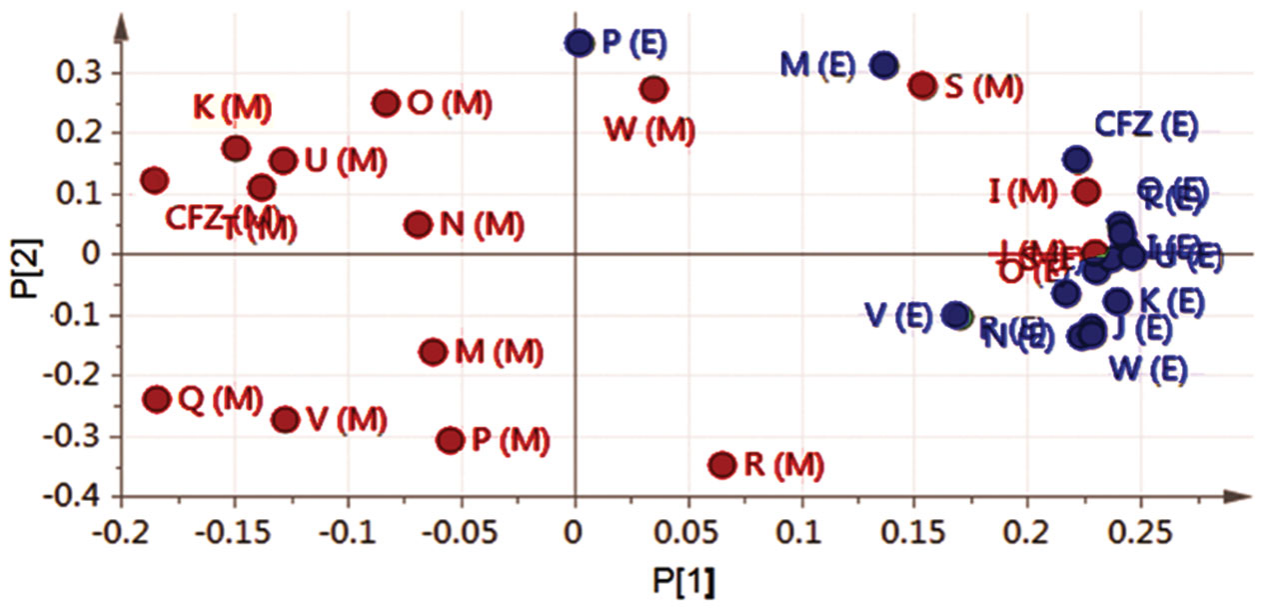
PCA confirms that the cell‐associated staining patterns of phenazine compounds corresponded with a greater variation in bioaccumulation and optical signals in macrophages. PCA was performed based on all optical properties analyzed for each phenazine compound in series 2 and 3, using five replicate measurements at 546 and 623 nm. In the plot, X(M) corresponds to Compound X in macrophages (red) and X(E) corresponds to Compound X in epithelial cells (blue). In this PCA plot, only the two main principal components are shown. These first two components of the PCA plot explained 97% of the variance in the data.

To establish the extent to which the differences in staining and self‐assembly of phenazine compounds may be due to cell type‐dependent differences in bioaccumulation, the total amount of compounds present in macrophages (**Figure** **6**a) and epithelial cells (Figure 6b) following an incubation period was measured and normalized by the number of cells. Overall, phenazine compounds tended to accumulate more in macrophages than in epithelial cells, regardless of ordered aggregate formation (Figure 6c). Nevertheless, compounds that exhibited the greatest accumulation in macrophages (Figure 6c,d) also yielded the most ordered inclusions (Compounds P, Q, U, V, and W). To confirm the cell type‐dependence of the bioaccumulation and intracellular self‐assembly properties of the phenazine compounds, principal component analysis (PCA) was performed on the cumulative bioaccumulation and diattenuation anisotropy data (**Figure** **7**). Because the first two components of the resulting PCA plots captured >97% of the variation in the measured properties of the compounds in macrophages and epithelial cells (Figure 7), the PCA plot indicates that most of the observed variation was associated with cell type specific differences in bioaccumulation and diattenuation anisotropy, without a clear association with chirality, chemical structure or the lipophilicity of the compounds.

**Figure 8:**
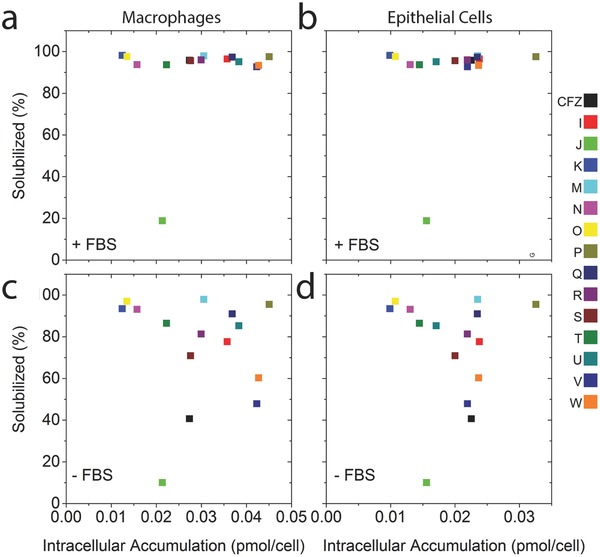
The solubility of phenazine compounds in cell culture media a,b) with FBS (+FBS) and c,d) without FBS (−FBS) plotted in relation to their cellular accumulation in a,c) RAW264.7 macrophages and b,d) MDCK epithelial cells.

Finally, we tested whether the bioaccumulation of the compounds was related to the precipitation of the compounds in cell culture media or to their solubility. In serum containing media, solutions of clofazimine and the other phenazine compounds were stably solubilized at the concentrations that were added to the cells, so the formation of extracellular precipitates is an unlikely explanation for the measured differences in bioaccumulation in either macrophages (**Figure** **8**a) or epithelial cells (Figure 8b). Only one of the phenazine analogs (Compound J) was not completely solubilized under these conditions. In the absence of serum (Figure 8c,d), the phenazine compounds exhibited significant variation in their solubility with some of the compounds precipitating in the media. However, the solubility of the compounds did not show a correlation with the measured bioaccumulation in macrophages (Figure 8c) or epithelial cells (Figure 8d).

Considering the biological mechanisms that may account for the observed variations in bioaccumulation properties of phenazine compounds, clofazimine and other small molecule drugs can be subject to chemical transformation by metabolic enzymes. Furthermore, small organic molecules can also be substrates of active transport mechanisms. These metabolic and active transport mechanisms are differentially expressed in macrophages and epithelial cells and they are highly specific and sensitive to the presence of different functional groups on the compounds.

## 3 Conclusion

To conclude, our results demonstrate that, in addition to clofazimine, other membrane‐permeant small molecule phenazine compounds can bioaccumulate and self‐assemble in macrophages, to a greater extent than in epithelial cells. Our results also demonstrate the use of monochromatic polarized light to measure diattenuation for monitoring the formation of ordered, insoluble complexes, which can be useful for assaying intracellular self‐assembly of phenazine compounds. This is an advantage of this approach compared to other types of optical techniques that have been developed to detect the presence of insoluble drug complexes inside cells.^16^ Indeed, with the quantitative polarization microscope, the presence of ordered molecular aggregates accompanying bioaccumulation of phenazine compounds was readily measurable and analyzable. Independently, we confirmed the bioaccumulation of the molecules by chemical analysis and the intracellular localization of the inclusions was confirmed with confocal microscopy by taking advantage of the compounds' fluorescence signals.

Similar to other structure–property relationship studies, our results demonstrate how the molecular organization of self‐assembling intracellular elements can be analyzed in relation to specific chemical features of the individual, small molecule building blocks. Based on staining patterns, the uptake of clofazimine was inhibited by replacing its chlorophenyl group with different aromatic functionalities. Notably, modification of the R‐imino group did not suppress intracellular inclusion formation. Instead, several R‐imino phenazine derivatives exhibited greater bioaccumulation than clofazimine, forming highly condensed cytoplasmic inclusions, which exhibited strong diattenuation anisotropy signals. In addition, biological factors affecting the preferential bioaccumulation of phenazine molecules in macrophages exerted the most dominant effect on self‐organization and intracellular inclusion formation, independently of the lipophilicity, solubility, and chirality of the molecules.

The condensation and phase separation of phenazine compounds into insoluble aggregates especially influences the bioaccumulation and retention of these compounds inside macrophages. In this regard, it is important to note that the measured concentration of phenazine compounds inside cells is far greater than one would expect from a nonspecific partitioning or a specific binding mechanism. For all the compounds that bioaccumulated inside cells, the measured numbers of phenazine molecules per cell (>0.03 ± 0.01 picomoles) exceeded the amounts of the most concentrated, endogenous biomolecules and metabolites (e.g., adenosine triphosphate^11^ or glutathione.^12^ Only potassium and water are expected to be present in greater amounts.^13^ Considering the possibility that bioaccumulation may reflect the partitioning of the molecules in membranes, there are in the order of 15 picograms of total lipids per leukocyte.^14^ This corresponds to 0.03 picomoles of total lipids per cell (calculated based on 500 g mol^−1^ of phospholipid). Interestingly, such intracellular precipitation behavior has been reported for other kinds of drugs.^15^ In fact, the size, faceted shapes, and overall morphology of these inclusions are inconsistent with the typical size, shapes and morphology of natural organelles^16^ and do not resemble the typical staining patterns of mitochondria^17^ or other cellular components stained with fluorescent probes.^18^ Therefore, one can infer that in addition to cell type‐specific differences in bioaccumulation, the propensity of poorly soluble phenazine molecules to aggregate into insoluble molecular complexes and phase separate from other cellular components also exerts an important influence on their intracellular disposition properties.

## 4 Experimental Section

*Materials for Chemical Synthesis*: The starting materials, reagents, and solvents for the synthesis were purchased from Sigma Aldrich, Fisher Scientific Acros, Oakwood Products or Chem‐Impex and used as such without purification. Biotage Initiator Classic, single‐mode Microwave Synthesizer was used for Microwave Syntheses. Compounds were purified by either Column Chromatography using Silicycle's SiliaFlash P60 (220–240 mesh) under positive house nitrogen pressure or Silicycle or Biotage prepacked flash columns using Biotage SP1 Flash System using two solvent gradient system. Solvent/Reagent Abbreviations or Formulae used: DCM, dichloromethane; EtOH, ethanol; EtOAc, ethyl acetate; DMSO, dimethylsulfoxide; MeCN, acetonitrile; AcOH, acetic acid; K_2_CO_3_, potassium carbonate; KF, potassium fluoride; Na_2_SO_4_, sodium sulfate; KOH, potassium hydroxide; FeCl_3_, ferric chloride; HCl, hydrochloric acid; Pd/C, palladium on carbon. NMR spectra were recorded on either Varian MR 400 MHz, or Varian Inova 500 MHz spectrometer. Chemical shifts were reported in *δ* (parts per million) in reference to the hydrogen peaks of tetramethylsilane, *δ* = 0.00. Mass spectra were recorded on a Micromass LCT Time‐of‐Flight instrument utilizing electrospray ionization in the positive ion mode (ESI^+^).

*Synthesis of Phenazine Derivatives*: Scheme 1 represents the overall synthesis procedures of the phenazine compounds. With the previously reported methods (using reagents, (a) KF/K_2_CO_3_ or (b) KOH/DMSO),^19^ aniline derivatives **1** and 2‐fluoronitrobenzene **2** were treated to produce the secondary amine derivatives **3** (*N*‐(4‐aryl)‐2‐nitroaniline). Using 10% Pd/C catalyst under hydrogen atmosphere, the nitro group in **3** was reduced to yield the diamine 4 (*N*‐arylbenzene‐1,2‐diamine). Then, derivative **4** was oxidized^20^ in aqueous ferric chloride solution to produce the corresponding phenazine salts **5** (3‐imino‐*N*,5‐bis(aryl)‐3,5‐dihydrophenazin‐2‐amine hydrochloride). Following treatments with various primary amines, the phenazine salts **5** yielded the corresponding phenazine derivatives **6** ((E)‐3‐(isopropylimino)‐*N*,5‐bis(aryl)‐3,5‐dihydrophenazin‐2‐amine) with 10%–85% yield.

*Absorbance Measurements*: Phenazine derivatives were solubilized at 0.1 mg mL^−1^ in methanol and in 9 M H_2_SO_4_. The UV–vis spectra of the different phenazine derivatives in methanol and in acidic solutions of H_2_SO_4_ were obtained in 96 well plates, using a Biotek microplate spectrophotometer.

*Physicochemical Property Predictions*: Estimation of various physicochemical properties of clofazimine and its chemical derivatives which could be important for predicting their behaviors when those chemicals are confronted by cellular/suborganellar membranes were made as follows: Clog P (the calculated logarithm of lipid/water partitioning coefficient of nonionic (neutral) forms of the compound) were calculated by Chemaxon software from Marvin Beans (http://www.chemaxon.com/marvin). Clog P values of ionized species were calculated using the procedures described by Hansch and Leo.^21^ Multiple p*K*_a_ (the dissociation constant of the protonated functional group) values (p*K*_a1_, p*K*_a2_, or p*K*_a3_) were calculated for the ionizable functional groups (amines) in these weakly basic molecules. Other structure parameters were estimated as described in the next section.^22^

*Predicting Cellular Uptake and Intracellular Localization of Phenazine Compounds*: Intracellular localization was predicted for each plausible ionic species of each phenazine compound as follow. For each species, structure parameter values, as estimated above, were inserted into published quantitative structure activity relationship (QSAR) models predicting cell uptake and intracellular localizations.^22^ The poor‐moderate‐good predictions in Tables S1, S3, and S5 (Supporting Information) relate to the Clog *P* of the major species present, usually the free base, as follows:

Poor: Clog *P* > 8 or Clog *P* < 0 [and/or number of rotatable bonds > 40]; moderate: 8 > Clog *P* > 5; Good: 5 > Clog *P* > 0.

Species close to prediction boundaries in parameter space are indicated by use of terms such as moderate‐good [i.e., the species falls into the moderate zone but close to the boundary with the good zone]. Predictions assume that p*K*_a_ values of basic groups are such that for most analogues a large proportion of each compound will be present in solution as a free base, with rather less as monocations, and even less as polycations, under physiological conditions. Polycations would only be present within acidic organelles. In Tables S1, S3, and S5 (Supporting Information): E = endoplasmic reticulum, C = cytosol, G = generic biomembranes, L = lysosome, M = mitochondrion, P = plasma membrane.

*Cell Culture*: RAW264.7 macrophages or MDCK (Madin Darby Canine Kidney) epithelial cells (strain II) were obtained from American Type Culture Collection (ATCC) (Manassas, VA) and cultured in 75 cm^2^ flasks at 37 °C, 5% CO_2_ containing humidified incubator. RAW264.7 macrophages (passage numbers 5–15) were grown in the medium containing Dulbecco's Modified Eagle Medium (DMEM (Gibco 11145); Invitrogen, Carlsbad, CA) with 2 × 10^−3^ m l‐glutamine, 4500 mg L^−1^ of d‐glucose, 110 mg L^−1^ of sodium pyruvate, 1% penicillin–streptomycin (Gibco 10378), and 10% fetal bovine serum (FBS; Gibco 10082). Confluent macrophage cells were detached by scraping and subcultured at 1:8 split ratios to culture flasks. MDCK cells (passage numbers 60–80) were cultured with growth medium consisting of DMEM supplemented with 1X nonessential amino acids (Gibco 11140), 1% penicillin–streptomycin, and 10% FBS. After reaching 70–80% confluence, MDCK cells were detached from the culture flasks using trypsin‐ethylenediaminetetracetic acid (trypsin‐EDTA) solution and subcultured at a split ratio of 1:5. Media in the flask was replaced every 3 d.

*Solubility Measurements*: Solutions containing the phenazine derivatives were made in cell culture media (DMEM) with or without FBS (10%). After a 24 h incubation, the solutions were centrifuged (10 000 × *g*, 10 min), the supernatant was removed and the precipitate was dissolved in 9 m H_2_SO_4_. The supernatant was diluted with 10 M NaOH to precipitate the remaining solubilized compound. The diluted supernatant was centrifuged (10 000 × *g*, 10 min) followed by dissolution of the precipitate in 9 m H_2_SO_4_. Both fractions were spectrophotometrically measured with a Biotek microplate spectrophotometer (*λ* = 540 nm) and phenazine content was determined using calibrated clofazimine standards.

*Cytotoxicity Measurements*: A 3′‐bis(4‐methoxy‐6‐nitro)benzene‐sulfonic acid hydrate (XTT) colorimetric assay was performed to assess the cytotoxicity of the clofazmine derivatives in RAW264.7 macrophages or MDCK epithelial cells using a Cell Proliferation Kit II (Roche Chemicals, Indianapolis, IN). Briefly, the cells were seeded in 96 well plates at a cell density of 8 × 10^3^ cells cm^−2^ (RAW264.7 macrophages) or 1.5 × 10^4^ cells cm^−2^ (MDCK epithelial cells) and after a 24 h incubation (37 °C, 5% CO_2_), cells were exposed to media containing various concentrations of clofazmine or its chemical derivatives (100 μL of 0.5 × 10^−6^, 1 × 10^−6^, 2 × 10^−6^, 4 × 10^−6^, 6 × 10^−6^, 8 × 10^−6^, 10 × 10^−6^, 15 × 10^−6^, 20 × 10^−6^, 25 × 10^−6^, 50 × 10^−6^, or 100 × 10^−6^ m) in DMEM (no phenol red; Gibco 21063) with 5% FBS and 1 × 10^−3^ m sodium pyruvate. After 72 h incubation with clofazmine or its derivatives, compound‐containing media was removed and cells were washed twice with media. The XTT labeling reagent (3′‐bis(4‐methoxy‐6‐nitro)benzene‐sulfonic acid hydrate) was freshly mixed with the electron coupling reagent (PMS: *N*‐methyl dibenzopyrazine methyl sulfate) before use according to the manufacturer's instructions. Cells were incubated in 100 μL of media (DMEM with 5% FBS) with XTT labeling mixture for 3 h (37 °C, 5% CO_2_). The absorbance value in each well was measured at 495 nm against a reference wavelength at 650 nm using a microplate reader (Synergy 2, BioTek Instruments, Winooski, VT). The experiments were repeated in three different sets for the various concentrations of compounds. The IC50 value for each compound was calculated from the concentration‐response curve generated by a nonlinear regression (curve fit) method in GraphPad Prism version 5.0 (GraphPad Software, Inc., San Diego, CA). The concentration of clofazimine or its chemical derivatives used for further in vitro cell studies was determined based on cell viability (%) assay results (see the next section).

*Cell Accumulation Experiments and Transmitted Light Microscopic Examination*: For cell cultures, RAW264.7 macrophages or MDCK epithelial cells were seeded in the 8‐well Nunc Lab‐Tek II chambered (#1.5) coverglasses (Thermo Scientific, Pittsburgh, PA) at a cell density of 8 × 10^3^ cells cm^−2^ or 1.5 × 10^4^ cells cm^−2^, respectively. After a 24 h incubation (37 °C, 5% CO_2_), cells were incubated with solution of clofazmine or its chemical derivatives (300 μL of 5 × 10^−6^ m concentration; measured cell viability ranging from 85% to 97% for all compounds tested at this concentration; Figure 1) in DMEM (no phenol red; plus 1 × 10^−3^ m sodium pyruvate and 5% FBS). For Compound “I,” 2.5 × 10^−6^ m of compound solution in media was used for the RAW 264.7 macrophages incubation because of lower cell viability at higher concentrations (IC50 = (3.95 ± 0.11) × 10^−6^ m). A solution of clofazmine or other chemical derivatives was made by a diluting stock solutions (2.5 × 10^−3^ m in DMSO; 10 × 10^−3^ m stock in DMSO were stored at −80 °C for further use) into DMEM with 5% FBS. Previously, we reported that clofazmine did not form visible precipitates in this media (DMEM with 5% FBS).^7^ For other phenazine derivatives, precipitates were avoided by including FBS in the media. The extracellular concentration of each compound in each well was maintained by daily replacement of the compound‐containing media. After 72 h of incubation with clofazmine or its derivatives, compound‐containing media were removed and cells were washed twice with media. Live cells in the chambered glasses were examined under inverted transmitted light microscopy (Nikon Eclipse T*i* microscope) with a 40× objective and color camera. As a control experiment, fixed cells were also microscopically examined after incubation with the compounds. Cells were seeded on the chambered cover glasses at the density of 1.5 × 10^4^ cells cm^−2^ (RAW264.7 macrophages) or 2.5 × 10^4^ cells cm^−2^ (MDCK epithelial cells). After an overnight incubation (37 °C, 5% CO_2_), media was removed from the wells and cold methanol (−20 °C) was added to each well. After 30 s, remaining methanol was removed by washing with media with 5% FBS. Fixed cells were incubated (37 °C, 5% CO_2_) with clofazmine or derivatives (300 μL of 5 × 10^−6^ m; 2.5 × 10^−6^ m for I) in media with 5% FBS. After 72 h, fixed RAW264.7 or MDCK cells were washed with media and examined using the same transmitted microscopy conditions as the live cells.

*Cell Associated Mass Measurements*: RAW264.7 macrophages or MDCK epithelial cells were seeded in 96 well plates at the density of 8 × 10^3^ cells cm^−2^ or 1.5 × 10^4^ cells cm^−2^, respectively. After an overnight incubation (37 °C, 5% CO_2_), cells were incubated with 100 μL of the media (DMEM with 5% FBS) containing 5 × 10^−6^ m clofazmine or its chemical derivatives (2.5 × 10^−6^ m of Compound “I”). Media containing each compound was replaced every day during the 72 h incubation. After the incubation, media was removed and cells were washed with Hank's balanced salt solution (HBSS) buffer (No. 14025; Invitrogen) twice. RAW264.7 macrophage cells in buffer were scraped and MDCK epithelial cells were exposed to Trypsin‐EDTA solution for detachment from the plates for cell number counting and quantification of cell‐associated masses. After centrifugation (650 × *g*), supernatant was removed and cell pellets were resuspended in 0.1 m citric acid/0.1 m trisodium citrate buffer (pH 5) for cell counting as previously reported.^7^ Equal numbers of cells in 100 μL in buffer were transferred to 96 well plates and 100 μL of detergent (ATCC, 30‐1010K) was added into wells for cell lysis. Chemical absorbance from each well was measured by the UV–vis microplate reader (Synergy 2, BioTek Instruments, Winooski, VT) at 490 nm wavelength and cell‐associated mass of each compound was calculated by a standard curve generated separately using the same media and presented as pmole/cell by normalizing the amount of chemical in each cell population sample by the number of cells in each sample.

*Linear Diattenuation Microscopy Instrument Set Up*: The LC‐PolScope was first developed as a birefringence imaging system at the Marine Biological Laboratory (Woods Hole, MA) by Oldenbourg and colleagues, which greatly increased the sensitivity and analytic power of the polarized light microscope.^9^ Recently, the technique was extended to include quantitative imaging of diattenuation and polarized fluorescence in biological and man‐made specimens.^10^, ^23^ For our studies of absorption properties of intracellular aggregates of phenazine derivatives, we used the diattenuation LC‐PolScope as reported earlier^10^ and described on the website OpenPolScope.org. The optical design was built on a Nikon Eclipse T*i* microscope equipped with the liquid crystal universal compensator consisting of a linear polarizer and a pair of liquid crystal devices. The LC compensator was part of the transillumination path and was used to illuminate the specimen with monochromatic, linearly polarized light of varying polarization orientation. There was no polarization analyzer present in the imaging path. Quantitative intensity images were recorded by a charge‐coupled device camera and a desktop computer calculated the average transmittance, the differential transmittance and the polarization orientation leading to maximum transmittance for each optically resolved picture element (pixel). Acquisition and processing steps were controlled using OpenPolScope plugins for the open source imaging programs ImageJ and Micro‐Manager (MMStudio version 1.4.15). With the combination of hardware and software, polarized light images were acquired at high sensitivity and high spatial resolution for measuring linear diattenuation.

*Linear Diattenuation Microscopy Data Acquisition and Analysis*: After RAW264.7 macrophages or MDCK epithelial cells were incubated with the compound in the chambered cover glasses for 72 h, at the same time of performing transmitted microscopic examination, cell specimens were examined under linear diattenuation microscopy using the Nikon Eclipse T*i* microscope with 40×/0.75 NA objective and monochromatic light of two different center wavelengths, 546 and 623 nm (passband 30 nm). In the OpenPolScope software, Pol‐Acquisition and Pol‐Analyzer plugins (version 2.0) were used to capture, process, and analyze the attenuation images. Before imaging the samples, the setup was calibrated using a specially prepared slide featuring four small pieces of linear polarization filters. The transmission axes of the filters were oriented in steps of 45° providing a means to calibrate the LC universal compensator settings. After the calibration, a sample slide was inserted and first a sequence of polarized light images of a clear, fully transmitting sample area was recorded, followed by an image sequence featuring the cells under investigation. The images of the clear area were used to determine instrument factors which were needed for computing the average and the polarization dependent loss in transmittance in cellular components visible in the second image.^10^

Linear diattenuation is a material property that can occur in materials such as crystals, in which the light absorbing molecules are arranged with a preferred orientation. The molecular alignment creates principal axes in the material denoting polarization directions for which light is maximally and minimally transmitted. Diattenuation is the difference between maximal (*T*_max_) and minimal (*T*_min_) transmittance, divided by their sum (Equation (1)):

(1) $$\mathrm{Diattenuation}=\frac{T_{\max}‐T_{\min}}{T_{\max}+T_{\min}}$$

To simultaneously measure the attenuation of many crystals with arbitrary orientations, we developed algorithms based on four specimen images, each recorded using light of a different polarization orientation: 0°, 45°, 90°, and 135°.^10^ The four raw images were used to calculate the average and differential transmittance and the orientation of maximum transmittance in every resolved image pixel. To isolate and analyze quantitative data of the objects (crystals), the anisotropy or maximum transmittance axis orientation image file opened in ImageJ was inverted (Ctrl + Shift + I) and thresholded using ImageJ's default automatic method (“Binary” → “Convert to Mask”). By using the ImageJ's algorithm, “Analyze Particles,” data of anisotropy or maximum transmittance axis orientation of objects in images could be obtained automatically. Diattenuation ratios were calculated by dividing anisotropy at 623 nm by that at 546 nm. For images obtained from cells incubated with Compounds, P, U, V, or W, maximum transmittance axis orientation lines in crystals were displayed by using OpenPolScope's plugin (Orientation‐LinesV3) installed in ImageJ.

*Confocal Fluorescent Microscopy*: A Nikon A1 confocal microscope was used for further investigation of live cells incubated with chiral pairs of compounds (T and U) and the parent compound, P. Since crystals or vesicular forms containing chemicals inside the cells incubated with Compound P, T, or U were fluorescent when viewed with the TRITC filter sets, intracellular distribution of these compounds in RAW264.7 or MDCK cells were visualized using a Nikon A1 confocal microscopy (Nikon Instruments Inc., Melville, NY) equipped with diode‐based lasers and a Nikon Apo 60×/1.4 NA oil immersion lens. Briefly, RAW264.7 macrophages or MDCK epithelial cells were seeded in the 8‐well Nunc Lab‐Tek II chambered (#1.5) cover glasses (Thermo Scientific, Pittsburgh, PA) at a density of 8 × 10^3^ cells cm^−2^ (RAW264.7 cells) or 1.5 × 10^4^ cells cm^−2^ (MDCK cells) and allowed to adhere overnight (37 °C, 5% CO_2_). Then, 5 × 10^−6^ m of Compound “P” or the related isomers, “T” or “U” in DMEM with 5% FBS (300 μL) was added to the cells and the cells were incubated for 72 h (37 °C, 5% CO_2_). For live cell imaging, after the removal of media, cells were washed with HBSS buffer twice. Hoechst 33342 (Invitrogen, Carlsbad, CA) was used for staining cell nuclei and FM 1‐43 (Molecular Probes T35356, Invitrogen) for the plasma membrane. Cells were incubated with 300 μL of 1:1 (v/v) dye mixtures of 5 μg mL^−1^ Hoechst 33342 and 7 × 10^−6^ m FM 1‐43 in HBSS for 15 min at room temperature. Without fixative, the confocal imaging of the live cells was performed using lasers for DAPI (excitation/emission wavelength: 405 nm/450 ± 25 nm), FITC (488 nm/525 ± 25 nm), and TRITC (561 nm/596 ± 25 nm) channels. Z‐stack images of the cells were captured along the Z‐axis (interval, 1 μm) in three fluorescence channels and analyzed by using the Nikon NIS‐Elements 3.2 confocal software (Nikon Instruments Inc., Melville, NY). In 3D reconstructions of the confocal sections assembled with the Nikon software, the cell nuclei fluoresced in the Hoechst/DAPI channel; the cell membranes fluoresced in the FITC channel; and, the intracellular inclusions fluoresced in the TRITC channel.

*Transmittance and Optical Density (OD) Calculations*: For calculating the differential transmittance of the inclusions, images at four different angles of polarization (0°, 45°, 90°, and 135°) were acquired by linear attenuation microscopy as described above. To measure transmittance, a threshold was applied to the mean transmitted image generated by the linear attenuation microscopy data acquisition. This threshold was determined using the ImageJ Auto‐thresholding protocol—Moments. Based on this threshold, a region of interest (ROI) image mask was generated, and then used to measure the mean transmittance (*T*_mean_) of the ROIs, expressed as mean pixel value. To generate transmittance image maps and for quantitative measurement of transmittance, pixel values were converted to absolute values by dividing the mean pixel value by 255 (8‐bit images). For the corresponding OD image maps and measurements, we used the following formula: OD = −log_10_(*T*_mean_). For each compound a total of five images were measured from experiments performed on three separate days (>200 cells analyzed per compound).

*Principal Component Analysis*: An “optical properties score” was calculated for each replicate measurement (*n* = 5) for each compound in each cell type (RAW264.7 macrophages and MDCK epithelial cells), by combining the anisotropy and optical density values at 546 and 623 nm wavelengths. PCA plots^24^ were generated using soft independent modelling in class analysis (SIMCA, http://www.umetrics.com/products/simca; Umeå, Sweden).

## Supporting information

As a service to our authors and readers, this journal provides supporting information supplied by the authors. Such materials are peer reviewed and may be re‐organized for online delivery, but are not copy‐edited or typeset. Technical support issues arising from supporting information (other than missing files) should be addressed to the authors.

Supplementary
