# Supplementary material for: Massive Bioaccumulation and Self‐Assembly of Phenazine Compounds in Live Cells
Source: Adv Sci (Weinh). 2015 Jun 5;2(8):1500025. doi: 10.1002/advs.201500025 (PMC4569013; doi:10.1002/advs.201500025)
Supplement: Supplementary file 1 — Supplementary [file ADVS-2-0e-s001.pdf]

Copyright WILEY-VCH Verlag GmbH & Co. KGaA, 69469 Weinheim, Germany, 2015.

ADVANCED SCIENCE
Open Access

Supporting Information

for *Adv. Sci.*, DOI: 10.1002/advs.201500025

Massive Bioaccumulation and Self-Assembly of Phenazine Compounds in Live Cells

*Kyoung Ah Min, Walajapet G. Rajeswaran, Rudolf Oldenbourg, Grant Harris, Rahul K. Keswani, Mason Chiang, Phillip Rzeczycki, Arjang Talattof, Mahwish Hafeez, Richard W. Horobin, Scott D. Larsen, Kathleen A. Stringer, and Gus R. Rosania**

Copyright WILEY-VCH Verlag GmbH & Co. KGaA, 69469 Weinheim, Germany, 2013.

Supporting Information

Title (Massive Bioaccumulation and Self-Assembly of Phenazine Compounds in Live Cells)

Kyoung Ah Min, Walajapet G. Rajeswaran, Rudolf Oldenbourg, Grant Harris, Rahul K. Keswani, Mason Chiang, Phillip Rzeczycki, Arjang Talattof, Mahwish Hafeez, Richard W. Horobin, Scott D. Larsen, Kathleen A. Stringer, and Gus R. Rosania

List of Contents:

1. Chemical Synthesis of **3** (N-(4-Aryl)-2-nitroaniline).
2. Chemical Synthesis of **4** (N-Arylbenzene-1,2-diamine).
3. Chemical Synthesis of **5** (3-Imino-N,5-bis(aryl)-3,5-dihydrophenazin-2-amine hydrochloride).
4. Chemical Synthesis of **6** ((E)-3-(Isopropylimino)-N,5-bis(aryl)-3,5-dihydrophenazin-2-amine).
5. General Procedures and Characterization Data of Riminophenazine Derivative Compounds.
6. Supplemental Results

1. Synthesis of **3** (N-(4-Aryl)-2-nitroaniline).
General Procedure A: K_2CO_3 (1.76 g, 12.8 mmole) and KF (0.49 g, 12.8 mmole) was mixed well by grinding in a mortar. This mixture was added to the 20 mL microwave reaction vial. Then aniline **1** (15.3 mmole, 1.2 equivalents), followed by fluoronitrobenzene **2** (1.35 mL, 12.8 mmole) were added and the reaction mixture was irradiated under a microwave at 180°C for a total of about 16 h and monitored by TLC at regular intervals. The solid mixture was extracted with DCM (4 × 20 mL), washed with 1N HCl (2 × 30 mL), water (2 × 50 mL), dried (Na_2SO_4) and the solvent was removed under reduced pressure to yield the crude product. It was purified using flash chromatography.
General Procedure B: To a solution of aniline **1** (10 mmole) and powdered KOH (2.81g, 50 mmole) in DMSO (10 mL) was added slowly fluoronitrobenzene (1.32 mL, 12.5 mmole) over a period of 0.5h. It was stirred at room temperature for 3h. The content was poured over ice and neutralized with 2N HCl. The precipitated orange solid was filtered, washed with water (200 mL), dried under suction and then under high vacuum overnight.

2. Synthesis of **4** (N-Arylbenzene-1,2-diamine).
General Procedure C: A 250-mL round bottom flask was flushed with N_2 and then charged with the nitro compound, **3**, MeCN (60 mL) and 10% Pd/C (0.11g). It was flushed again with N_2 and sealed with rubber septum. The evacuated flask was filled with H_2 and it was stirred at RT in a H_2 atmosphere using a balloon over night. TLC results after overnight stirring indicated completion of reaction. The solution was filtered with the aid of celite. The solvent was removed under reduced pressure to yield the compound **4**. It was purified if necessary.

3. Synthesis of **5** (3-Imino-N,5-bis(aryl)-3,5-dihydrophenazin-2-amine hydrochloride).
General Procedure D: To a solution of the diamino compound (2.9 mmole) in glacial AcOH (4.5 mL) was added a solution of $FeCl_3.6H_2O$ (8.7 mmole, 3 equivalent) in water (15 mL) and 12 N HCl (0.4 mL, 4.5 equivalent). The reaction was left stirring at room temperature overnight after dilution with 7 mL of H_2O. The reaction was further diluted with 10 mL of H_2O. After 1h, the precipitate was filtered, washed with excess water, dried under suction and then inside a vacuum desiccator.

4. Synthesis of **6** ((E)-3-(Isopropylimino)-N,5-bis(aryl)-3,5-dihydrophenazin-2-amine).
General Procedure E: The phenazine hydrochloride (0.1g) was suspended in dioxane or EtOH (1 mL). To this suspension was added isopropylamine (0.2 mL) and the mixture was heated in a sealed tube for 5 h. The solution was filtered and the flask was washed with EtOH (1 mL). The filtrate was diluted with H_2O until it becomes slightly turbid. The compound was crystallized and then recrystallized from aqueous EtOH or purified by the flash chromatography.

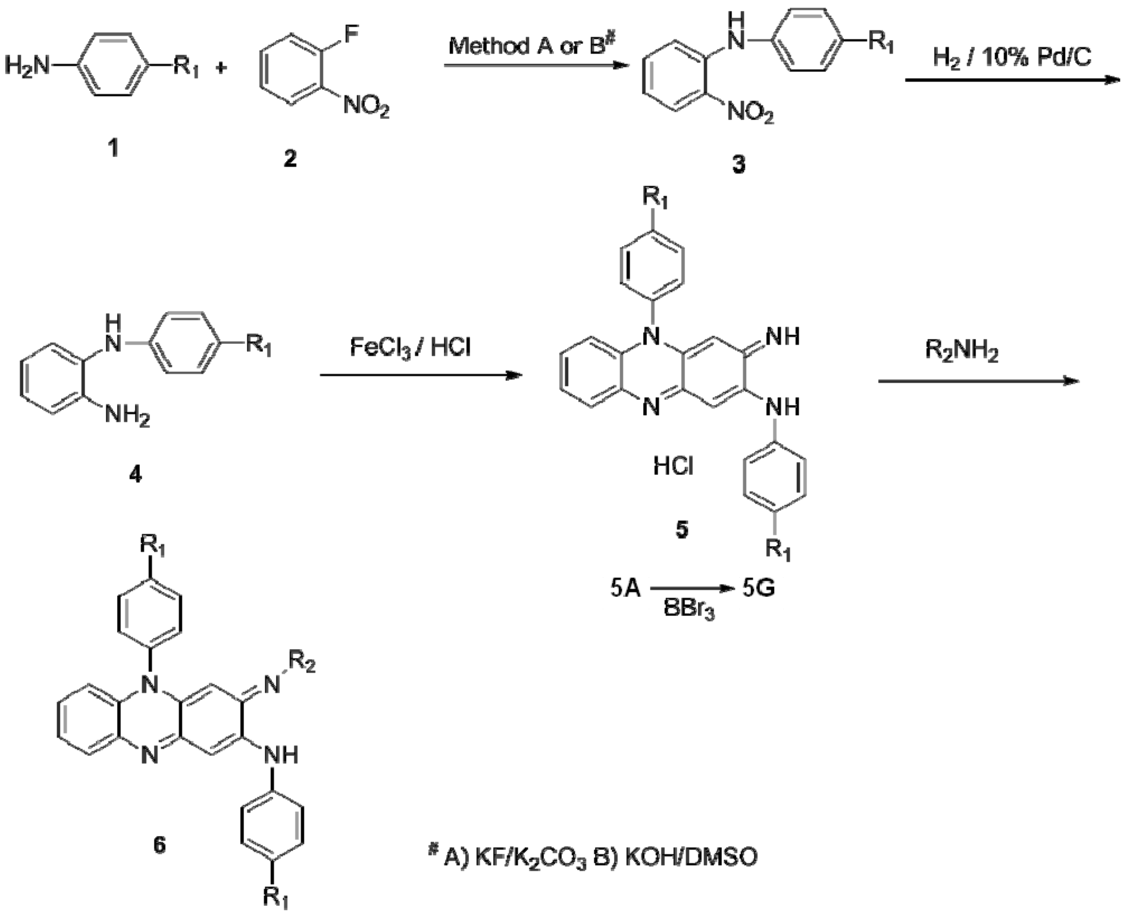

Figure S1. Synthesis scheme for phenazine compounds. Treatment of aniline derivatives **1** with 2-fluoronitrobenzene **2** following the published procedure[12] (Method A using KF/K_2CO_3 or B using KOH/DMSO) gave the secondary amine derivatives **3** in 22 - 63% yield. Reduction of the nitro group was carried out using 10% Pd/C catalyst under hydrogen atmosphere to yield the diamine **4** in 55 - 99% yield. Then the diamine **4** was oxidised[3, 5] in aqueous ferric chloride solution to give the corresponding phenazine salts **5** in 70 - 96%. The dimethoxyphenazine salt **5A** was demethylated using borontribromide in dichloromethane to give the hydroxyphenazine hydrobromide **5G** in 67% yield. The phenazine salts **5** on treatment with variety of primary amines gave the corresponding phenazine derivatives **6** in 10 - 85% yield.

5. General procedures and characterization data of riminophenazine derivative compounds.

A (6-206566)

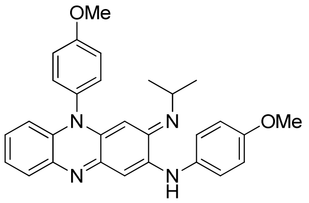

N-(4-Methoxyphenyl)-2-nitroaniline, **3A** (General Procedure A): Purified by the flash chromatography using 5-40% EtOAc/hexanes. Yield: 1.25g (63%); ^{1}H NMR (500 MHz, $CDCl_3$) δ 9.41 (s, 1H), 8.21 (d, *J* = 8.5 Hz, 1H), 7.35 – 7.31 (m, 1H), 7.23 – 7.19 (m, 2H), 7.03 – 7.00 (m, 1H), 6.99 – 6.95 (m, 2H), 6.75 – 6.70 (m, 1H), 3.86 (s, 3H).
N-(4-Methoxyphenyl)benzene-1,2-diamine, **4A** (General Procedure C): Purified using 5-40% EtOAc/Hexanes gradient Yield: 0.62g (59%); ^{1}H NMR (500 MHz, $CDCl_3$) δ 7.06 – 7.00 (m, 2H), 6.82 – 6.78 (m, 2H), 6.76 – 6.70 (m, 3H), 6.67 (td, *J* = 7.5, 1.4 Hz, 1H), 3.77 (s, 3H), 3.17 (q, *J* = 7.2 Hz, 2H) & 1.24 (t, *J* = 7.2 Hz, 3H).
3-Imino-N,5-bis(4-methoxyphenyl)-3,5-dihydrophenazin-2-amine hydrochloride, **5A** (General Procedure D): Yield: 1.2g (90%); MS (ESI$^+$), *m/z*: 423.2 (M+1); ^{1}H NMR (500 MHz, CD_3OD) δ 8.21 – 8.16 (m, 1H), 7.81 – 7.72 (m, 3H), 7.56 (d, *J* = 1.1 Hz, 1H), 7.44 – 7.38 (m, 2H), 7.25 – 7.21 (m, 2H), 7.18 – 7.14 (m, 1H), 7.03 – 7.00 (m, 1H), 6.98 –6.95 (m, 1H), 6.86 – 6.83 (m, 1H), 6.39 (s, 1H), 3.91 (s, 3H) & 3.85 (s, 3H).
(E)-3-(Isopropylimino)-N,5-bis(4-methoxyphenyl)-3,5-dihydrophenazin-2-amine,[1] **6A** (General Procedure E): Yield: 67 mg (66%); MS (ESI$^+$), *m/z*: 465.2 (M+1); ^{1}H NMR (400 MHz, $CDCl_3$) δ 7.67 (d, *J* = 7.6 Hz, 1H), 7.60 (t, *J* = 8.1 Hz, 1H), 7.23 (d, *J* = 6.7 Hz, 1H), 7.17 – 7.07 (m, 3H), 6.98 (d, *J* = 7.9 Hz, 1H), 6.93 – 6.81 (m, 4H), 6.62 (d, *J* = 8.2, 2.4 Hz, 1H), 6.52 (d, *J* = 8.3 Hz, 1H), 5.33 (s, 1H), 3.83 (d, *J* = 11.8 Hz, 6H), 3.44 (h, *J* = 6.3 Hz,1H) & 1.07 (t, *J* = 6.5 Hz, 6H).

B (8-206567)

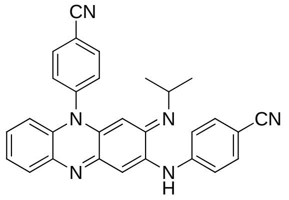

4-((2-Nitrophenyl)amino)benzonitrile, **3B** (General Procedure A): Purified by the flash chromatography using 5-40% EtOAc/hexanes. Yield: 0.84g (28%); ^{1}H NMR (500 MHz, $CDCl_3$) δ 9.43 (s, 1H), 8.23 (dd, *J* = 8.6, 1.5 Hz, 1H), 7.66 (d, *J* = 8.2 Hz, 2H), 7.54 – 7.44 (m, 2H), 7.33 (d, *J* = 8.7 Hz, 2H), 7.02 – 6.95 (m, 1H).
4-((2-Aminophenyl)amino)benzonitrile, **4B** (General Procedure C): Yield: 0.38g (99%); ^{1}H NMR (500 MHz, $CDCl_3$) δ 7.45 (d, *J* = 8.5 Hz, 2H), 7.17 – 7.07 (m, 2H), 6.84 (d, *J* =7.8 Hz, 1H), 6.82 – 6.78 (m, 1H), 6.68 (d, *J* = 8.5 Hz, 2H), 5.62 (s, 1H) & 3.79 (s, 2H).
4-((5-(4-Cyanophenyl)-3-imino-3,5-dihydrophenazin-2-yl)amino)benzonitrile hydrochloride, **5B** (General Procedure D): Yield: 0.33g (85%); MS (ESI$^+$), *m/z*:: 413 (M+1); ^{1}H NMR (500 MHz, $(CD_3)_2SO$) δ 10.13 (s, 1H), 9.56 – 9.37 (m, 2H), 8.45 (d, *J* = 8.1 Hz, 2H), 8.30 – 8.23 (m, 1H), 7.99 (d, *J* = 8.0 Hz, 2H), 7.88 (d, *J* = 8.3 Hz, 2H), 7.82 – 7.77 (m, 2H), 7.71 (s, 1H), 7.62 (d, *J* = 8.3 Hz, 2H), 7.10 – 7.05 (m, 1H), 6.18 (s, 1H).
(E)-4-((5-(4-Cyanophenyl)-3-(isopropylimino)-3,5-dihydrophenazin-2-yl)amino)benzonitrile, **6B** (General Procedure E): Yield: 49 mg (48%); MS (ESI$^+$), *m/z*: 455.2 (M+1); ^{1}H NMR (400

MHz, $CDCl_3$) δ 8.06 (d, *J* = 8.1 Hz, 2H), 7.73 (d, *J* = 7.8 Hz, 1H), 7.63 (d, *J* = 8.3 Hz, 2H), 7.53 (d, *J* = 8.1 Hz, 2H), 7.41 (d, *J* = 8.4 Hz, 2H), 7.24 – 7.14 (m, 2H), 7.03 (s, 1H), 6.37 (d, *J* = 8.1 Hz, 1H), 5.18 (s, 1H), 3.41 (p, *J* = 6.1 Hz, 1H), 1.08 (d, *J* = 6.2 Hz, 6H).

C (12-206693)

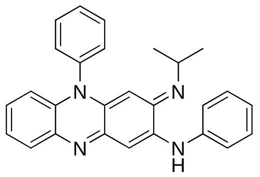

2-Nitro-N-phenylaniline, **3C** (General Procedure A): Purified by the flash chromatography using 3% EtOAc/hexanes. Yield: 0.52g (23%); ^{1}H NMR (400 MHz, $CDCl_3$) δ 9.49 (s, 1H), 8.20 (dd, *J* = 8.6, 1.7 Hz, 1H), 7.44 – 7.34 (m, 3H), 7.30 – 7.20 (m, 4H), 6.77 (ddd, *J* = 8.4, 6.9, 1.4 Hz, 1H).
N-Phenylbenzene-1,2-diamine, **4C** (General Procedure C): Yield: 0.41g (97%); ^{1}H NMR (400 MHz, $CDCl_3$) δ 7.26 – 7.17 (m, 2H), 7.12 (dd, *J* = 7.8, 1.5 Hz, 1H), 7.06 – 6.97 (m, 1H), 6.86 – 6.71 (m, 5H), 5.17 (s, 1H), 3.76 (s, 2H).
3-Imino-N,5-diphenyl-3,5-dihydrophenazin-2-amine hydrochloride, **5C** (General Procedure D): Yield: 0.32g (74%); ^{1}H NMR (400 MHz, $(CD_3)_2SO$) δ 9.67 (s, 1H), 9.38 (s, 2H), 8.21 – 8.14 (m, 1H), 7.95 – 7.83 (m, 3H), 7.77 – 7.70 (m, 4H), 7.55 – 7.46 (m, 4H), 7.34 (s, 1H), 7.29 – 7.24 (m, 1H), 7.02 – 6.95 (m, 1H), 6.21 (s, 1H).
(E)-3-(Isopropylimino)-N,5-diphenyl-3,5-dihydrophenazin-2-amine,[1a] **6C** (General Procedure E): Yield: 65 mg (85%); ^{1}H NMR (400 MHz, $CDCl_3$) δ 7.76 – 7.59 (m, 4H), 7.49 – 7.25 (m, 7H), 7.17 – 7.04 (m, 3H), 6.89 (s, 1H), 6.45 (d, *J* = 8.1 Hz, 1H), 5.25 (s, 1H), 3.39 (d, *J* = 6.2 Hz, 1H), 1.04 (d, *J* = 6.2 Hz, 6H).

D (4-206694)

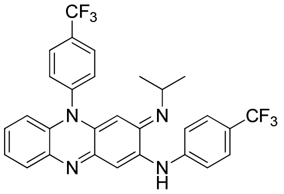

2-Nitro-N-(4-(trifluoromethyl)phenyl)aniline, **3D** (General Procedure A): Purified by the flash chromatography using 15-65% EtOAc/hexanes. Yield: 0.15g (7%); ^{1}H NMR (400 MHz, $CDCl_3$) δ 9.47 (s, 1H), 8.23 (d, *J* = 8.5 Hz, 1H), 7.65 (d, *J* = 8.3 Hz, 2H), 7.48 – 7.43 (m, 1H), 7.40 – 7.34 (m, 3H), 6.93 – 6.87 (m, 1H).
N-(4-(Trifluoromethyl)phenyl)benzene-1,2-diamine, **4D** (General Procedure C): Yield: 0.13g (99%); ^{1}H NMR (400 MHz, $CDCl_3$) δ 7.41 (d, *J* = 8.6 Hz, 2H), 7.14 – 7.03 (m, 2H), 6.85 – 6.72 (m, 2H), 6.70 (d, *J* = 8.6 Hz, 2H), 5.41 (s, 1H), 3.76 (s, 2H).
3-Imino-N,5-bis(4-(trifluoromethyl)phenyl)-3,5-dihydrophenazin-2-amine hydrochloride, **5D** (General Procedure D): Yield: 0.12g (94%); ^{1}H NMR (400 MHz, $(CD_3)_2SO$) δ 9.75 (s, 1H), 9.40 – 9.22 (m, 2H), 8.34 (d, *J* = 8.1 Hz, 2H), 8.27 – 8.23 (m, 1H), 8.22 – 8.17 (m, 1H), 8.01 (d, *J* = 8.1 Hz, 2H), 7.96 – 7.90 (m, 1H), 7.85 – 7.77 (m, 4H), 7.73 – 7.68 (m, 1H), 7.66 – 7.62 (m, 3H), 7.47 (t, *J* = 8.1 Hz, 1H), 7.28 (s, 1H), 7.05 (dd, *J* = 7.0, 3.0 Hz, 1H), 6.18 (s, 1H).
(*E*)*-3-(Isopropylimino)-N,5-bis(4-(trifluoromethyl)phenyl)-3,5-dihydrophenazin-2-amine*, **6D** (General Procedure E): Yield: 52 mg (52%); MS (ESI$^+$), *m/z*: 541.1 (M+1); ^{1}H NMR (400 MHz, $CDCl_3$) δ 8.02 (d, *J* = 8.2 Hz, 2H), 7.72 (dd, *J* = 7.8, 1.3 Hz, 1H), 7.61 (d, *J* = 8.4 Hz,

2H), 7.53 (d, J = 8.1 Hz, 2H), 7.44 (d, J = 8.4 Hz, 2H), 7.23 – 7.12 (m, 2H), 7.00 (s, 1H), 6.39 (d, J = 7.9 Hz, 1H), 5.22 (s, 1H), 3.42 (hept, J = 6.3 Hz, 1H), 1.08 (d, J = 6.2 Hz, 6H), 9.07 – 8.48 (m, 1H).

E (11-206695)

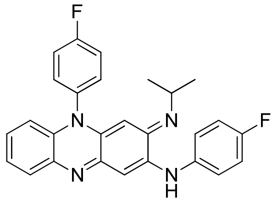

N-(4-Fluorophenyl)-2-nitroaniline, **3E** (General Procedure B): Yield: 0.52g (22%); ^{1}H NMR (400 MHz, $CDCl_3$) δ 9.40 (s, 1H), 8.21 (dd, J = 8.6, 1.6 Hz, 1H), 7.40 – 7.33 (m, 1H), 7.25 (dd, J = 9.8, 3.8 Hz, 2H), 7.12 (t, J = 8.5 Hz, 2H), 7.05 (dd, J = 8.6, 1.3 Hz, 1H), 6.81 – 6.74 (m, 1H).
N-(4-Fluorophenyl)benzene-1,2-diamine, **4E** (General Procedure C): Purified using 12-15% EtOAc/Hexanes gradient. Yield: 0.36g (79%); ^{1}H NMR (400 MHz, $CDCl_3$) δ 7.05 (dd, J = 7.8, 1.5 Hz, 1H), 7.02 – 6.97 (m, 1H), 6.95 – 6.87 (m, 2H), 6.83 – 6.64 (m, 4H), 5.06 (s, 1H), 3.74 (s, 2H).
3-Imino-N,5-bis(4-fluorophenyl)-3,5-dihydrophenazin-2-amine hydrochloride, **5E** (General Procedure D): Yield: 0.36g (96%); ^{1}H NMR (500 MHz, $(CD_3)_2SO$) δ 9.56 (s, 1H), 9.41 – 9.17 (m, 2H), 8.18 (d, J = 7.3 Hz, 1H), 7.84 – 7.71 (m, 6H), 7.52 – 7.47 (m, 2H), 7.41 – 7.34 (m, 2H), 7.25 – 7.17 (m, 1H), 7.07 (d, J = 7.6 Hz, 1H), 6.24 – 6.19 (m, 1H).
(E)-N,5-bis(4-Fluorophenyl)-3-(isopropylimino)-3,5-dihydrophenazin-2-amine,[1b] **6E** (General Procedure E): Yield: 90 mg (59%); MS (ESI$^+$), *m/z*: 441.1 (M+1); ^{1}H NMR (400 MHz, $CDCl_3$) δ 7.67 (dd, J = 7.8, 1.7 Hz, 1H), 7.45 – 7.38 (m, 2H), 7.36 – 7.29 (m, 4H), 7.20 – 7.02 (m, 5H), 6.71 (s, 1H), 6.45 (d, J = 8.0 Hz, 1H), 5.26 (s, 1H), 3.45 (hept, J = 6.4 Hz, 1H), 1.08 (d, J = 6.3 Hz, 6H).

F (9-206696)

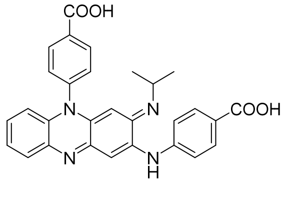

4-((2-Nitrophenyl)amino)benzoic acid, **3F** (General Procedure B): Yield: 1.55g (60%) (It's a mixture of about 1:3 ester/carboxylic acid and used in subsequent transformation as such without further purification); ^{1}H NMR (500 MHz, $(CD_3)_2SO$) δ 12.64 (s, 1H), 9.31 (s, 1H), 8.16 – 8.04 (m, 1H), 7.93 – 7.84 (m, 2H), 7.63 – 7.44 (m, 2H), 7.38 – 7.26 (m, 2H), 7.13 – 7.00 (m, 1H).
4-((2-Aminophenyl)amino)benzoic acid, **4F** (General Procedure C): Crystallized from aqueous Methanol. Yield: 0.24g (55%); ^{1}H NMR (500 MHz, $(CD_3)_2SO$) δ 12.17 (s, 1H), 7.86 (d, J = 28.3 Hz, 1H), 7.69 (d, J = 8.7 Hz, 2H), 7.01 (d, J = 7.8 Hz, 1H), 6.96 – 6.89 (m, 1H), 6.79 – 6.74 (m, 1H), 6.65 (d, J = 8.7 Hz, 2H), 4.95 – 4.69 (m, 2H), 6.61 – 6.53 (m, 1H).
4-((5-(4-Carboxyphenyl)-3-imino-3,5-dihydrophenazin-2-yl)amino)benzoic acid hydrochloride, **5F** (General Procedure D): Yield: 0.17g (70%); ^{1}H NMR (400 MHz, $(CD_3)_2SO$) δ 13.05 (s, 2H), 9.76 (s, 1H), 9.33 (s, 2H), 8.45 – 8.40 (m, 2H), 8.28 – 8.23 (m, 1H), 8.03 (d, J = 8.5 Hz, 2H), 7.87 (d, J = 8.4 Hz, 2H), 7.82 – 7.74 (m, 2H), 7.68 – 7.64 (m, 1H), 7.55 (d, J = 8.1 Hz, 2H), 7.11 – 7.03 (m, 1H), 6.23 – 6.19 (m, 1H).

(E)-4-((5-(4-Carboxyphenyl)-3-(isopropylimino)-3,5-dihydrophenazin-2-yl)amino)benzoic acid, **6F** (General Procedure E): Yield: 90 mg (59%); MS(ESI^+), *m/z*: 494.1 (M+1); ^{1}H NMR (400 MHz, CD_3OD) δ 8.38 (d, *J* = 8.0 Hz, 2H), 8.27 – 8.19 (m, 1H), 8.07 (d, *J* = 8.2 Hz, 2H), 7.81 – 7.72 (m, 2H), 7.69 (s, 1H), 7.59 (d, *J* = 7.9 Hz, 2H), 7.40 (d, *J* = 8.2 Hz, 2H), 7.26 – 7.16 (m, 1H), 6.34 (s, 1H), 1.28 (d, *J* = 6.6 Hz, 6H).

G (5-206608)

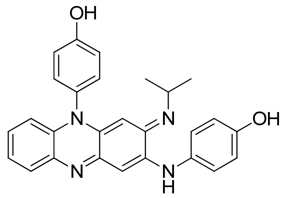

Method for chemical synthesis of 4-((5-(4-Hydroxyphenyl)-3-imino-3,5-dihydrophenazin-2-yl)amino)phenol hydrobromide, **5G:** To the solution of the phenazine **5A** (56 mg) in DCM (3 mL) was added BBr_3 in DCM (1 mL) and the solution was stirred at RT for 4 days. Water (4 mL) was added to quench the reaction, the precipitate was filtered, washed with water (15 mL) and dried under vacuum desiccator for 2 days. Yield: 39 mg (67%); MS (ESI^+), *m/z*: 395.1 (M+1); 1H NMR (400 MHz, $(CD_3)_2SO$) δ 10.38 (s, 1H), 9.74 (s, 1H), 9.27 (s, 1H), 8.70 (s, 1H), 8.20 (s, 1H), 7.81 – 7.64 (m, 3H), 7.42 (s, 1H), 7.34 – 7.19 (m, 2H), 7.16 – 7.05 (m, 3H), 6.85 (s, 2H), 6.73 – 6.60 (m, 1H), 6.31 (s, 1H).

(E)-4-((5-(4-Hydroxyphenyl)-3-(isopropylimino)-3,5-dihydrophenazin-2-yl)amino)phenol, **6G** (General Procedure E): Purified using 40-100% EtOAc/Hexanes gradient. Yield: 19 mg (65%); MS (ESI^+), *m/z*: 437.2 (M+1); ^{1}H NMR (400 MHz, $(CD_3)_2SO$) δ 10.03 (s, 1H), 9.50 (s, 1H), 7.60 (d, *J* = 7.5 Hz, 1H), 7.57 – 7.51 (m, 1H), 7.21 – 7.00 (m, 5H), 6.84 (d, *J* = 7.9 Hz, 1H), 6.82 – 6.80 (m, 1H), 6.78 – 6.74 (m, 2H), 6.72 – 6.69 (m, 1H), 6.49 (t, *J* = 8.3 Hz, 2H), 5.26 (s, 1H), 3.33 (q, *J* = 6.8 Hz, 1H), 1.03 – 0.98 (m, 6H).

H (1-206570)

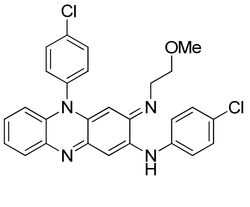

(E)-N,5-bis(4-chlorophenyl)-3-((2-methoxyethyl)imino)-3,5-dihydrophenazin-2-amine,[1a] **6H** (General Procedure E): Purified using 10-50% EtOAc/Hexanes gradient. Yield: 18 mg (17%); MS (ESI^+), *m/z*: 490.1 & 492.1 (M+1); ^{1}H NMR (400 MHz, $CDCl_3$) δ 7.74 – 7.67 (m, 3H), 7.38 – 7.26 (m, 7H), 7.24 – 7.09 (m, 3H), 6.84 (s, 1H), 6.50 – 6.44 (m, 1H), 5.28 (s, 1H), 3.72 (t, *J* = 6.2 Hz, 2H), 3.40 (s, 3H), 3.31 (t, *J* = 6.2 Hz, 2H).

I (7-206568)

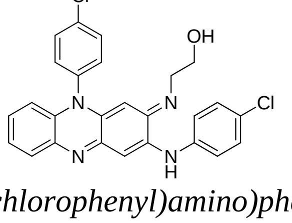

(E)-2-((10-(4-Chlorophenyl)-3-((4-chlorophenyl)amino)phenazin-2(10H)-ylidene)amino)ethanol ,[2] **6I** (General Procedure E): Yield: 41 mg (40%); MS (ESI^+), *m/z*: 475.1 & 477.1 (M+1); ^{1}H NMR (400 MHz, $CDCl_3$) δ 7.74 – 7.65 (m, 3H), 7.33 – 7.28 (m,

2H), 7.27 – 7.21 (m, 5H), 7.21 – 7.07 (m, 3H), 6.83 (s, 1H), 6.47 (d, *J* = 7.7 Hz, 1H), 5.25 (s, 1H), 3.88 (t, *J* = 5.3 Hz, 2H), 3.23 (t, *J* = 5.3 Hz, 2H).

J (13-206689)

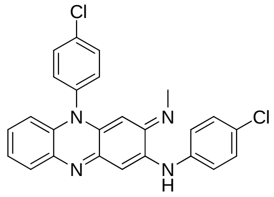

(E)-N,5-Bis(4-chlorophenyl)-3-(methylimino)-3,5-dihydrophenazin-2-amine,[2] **6J** (General Procedure E): Purified using 15 - 60% EtOAc/Hexanes gradient. Yield: 28 mg (29%); MS (ESI^+), *m/z*: 446.1 & 448.1 (M+1); ^{1}H NMR (400 MHz, $CDCl_3$) δ 7.69 (d, *J* = 8.6 Hz, 3H), 7.35 – 7.24 (m, 7H), 7.20 – 7.11 (m, 2H), 6.83 (s, 1H), 6.46 (d, *J* = 8.1 Hz, 1H), 5.27 (s, 1H), 3.06 (s, 3H).

K (3-206690)

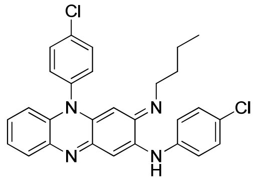

(E)-3-(Butylimino)-N,5-bis(4-chlorophenyl)-3,5-dihydrophenazin-2-amine,[2] **6K** (General Procedure E): Purified using 35 - 45% EtOAc/Hexanes gradient. Yield: 38 mg (37%); MS (ESI^+), *m/z*: 487.1 & 489.1 (M+1); ^{1}H NMR (400 MHz, $CDCl_3$) δ 7.72 – 7.64 (m, 3H), 7.32 – 7.25 (m, 6H), 7.19 – 7.08 (m, 3H), 6.81 (s, 1H), 6.44 (d, *J* = 8.0 Hz, 1H), 5.24 (s, 1H), 3.14 (t, *J* = 6.9 Hz, 2H), 1.61 – 1.55 (m, 2H), 1.38 – 1.31 (m, 2H), 0.87 (t, *J* = 7.3 Hz, 3H).

L (15-208923)

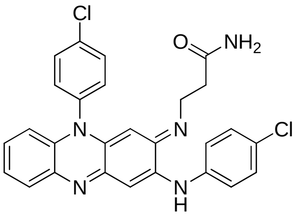

(E)-3-((10-(4-Chlorophenyl)-3-((4-chlorophenyl)amino)phenazin-2(10H)-ylidene)amino)-propanamide, **6L** (General Procedure E): Yield: 18 mg (24%); MS(ESI^+), *m/z*: 502.0 & 504.0 (M+1); ^{1}H NMR (400 MHz, $CDCl_3$) δ 7.77 (d, *J* = 7.9 Hz, 1H), 7.71 (d, *J* = 8.6 Hz, 2H), 7.34 – 7.25 (m, 8H), 7.23 – 7.18 (m, 2H), 6.91 (s, 1H), 6.54 (d, *J* = 8.3 Hz, 1H), 5.32 (s, 2H), 3.38 (t, *J* = 6.2 Hz, 2H), 2.65 (t, *J* = 6.2 Hz, 2H).

M (16-208922)

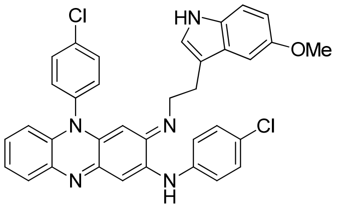

(E)-N,5-Bis(4-chlorophenyl)-3-((2-(5-methoxy-1H-indol-3-yl)ethyl)imino)-3,5-dihydrophenazin-2-amine, **6M** (General Procedure E): Yield: 26 mg (20%); MS(ESI^+), *m/z*: 604.0 & 606.0 (M+1); ^{1}H NMR (400 MHz, $CDCl_3$) δ 7.84 (s, 1H), 7.69 (d, *J* = 8.1 Hz, 1H), 7.62 (d, *J* = 8.4 Hz, 2H), 7.32 (d, *J* = 8.7 Hz, 2H), 7.25 – 7.21 (m, 3H), 7.20 – 7.09 (m, 5H),

7.06 – 7.04 (m, 1H), 6.90 – 6.83 (m, 3H), 6.41 (d, *J* = 8.1 Hz, 1H), 5.19 (s, 1H), 3.82 (s, 3H), 3.49 (t, *J* = 7.0 Hz, 2H), 3.07 (t, *J* = 7.0 Hz, 2H).

N (17-208921)

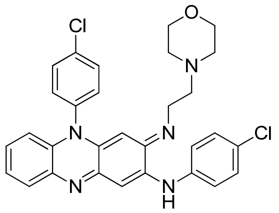

(E)-N,5-Bis(4-chlorophenyl)-3-((2-morpholinoethyl)imino)-3,5-dihydrophenazin-2-amine,[3] **6N** (General Procedure E): Yield: 38 mg (33%); MS(ESI$^+$), *m/z*: 544.0 & 546.0 (M+1); ^{1}H NMR (400 MHz, $CDCl_3$) δ 7.72 – 7.66 (m, 2H), 7.36 – 7.24 (m, 7H), 7.23 – 7.06 (m, 3H), 6.84 (s, 1H), 6.45 (d, *J* = 8.2 Hz, 1H), 5.24 (s, 1H), 3.74 – 3.65 (m, 4H), 3.33 (t, *J* = 7.3 Hz, 2H), 2.68 (t, *J* = 7.3 Hz, 2H), 2.50 – 2.40 (m, 4H).

O (19-209042)

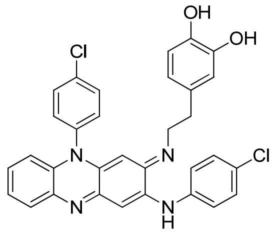

(E)-4-(2-((10-(4-Chlorophenyl)-3-((4-chlorophenyl)amino)phenazin-2(10H)-ylidene)amino)-ethyl)benzene-1,2-diol, **6O** (General Procedure E): Yield: 13 mg (17%); MS(ESI$^+$), *m/z*: 567.0 & 569.0 (M+1); ^{1}H NMR (400 MHz, CD_3OD) δ 8.22 – 8.17 (m, 1H), 7.98 – 7.92 (m, 2H), 7.78 – 7.73 (m, 2H), 7.59 – 7.55 (m, 2H), 7.51 (s, 1H), 7.48 – 7.45 (m, 2H), 7.36 – 7.32 (m, 2H), 7.17 – 7.13 (m, 1H), 6.67 (d, *J* = 7.9 Hz, 1H), 6.55 – 6.52 (m, 1H), 6.28 – 6.22 (m, 1H), 5.89 (s, 1H), 3.47 (d, *J* = 7.3 Hz, 2H), 2.72 (t, *J* = 7.3 Hz, 2H).

P (10-206691)

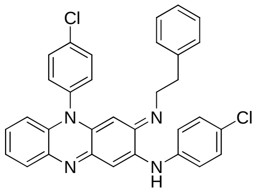

(E)-N,5-Bis(4-chlorophenyl)-3-(phenethylimino)-3,5-dihydrophenazin-2-amine,[1b, 4] **6P** (General Procedure E): Yield: 53 mg (46%); MS (ESI$^+$), *m/z*: 535.0 & 537.0 (M+1); ^{1}H NMR (400 MHz, $CDCl_3$) δ 7.75 – 7.65 (m, 3H), 7.37 – 7.26 (m, 8H), 7.23 – 7.16 (m, 3H), 7.15 – 7.09 (m, 3H), 6.86 (s, 1H), 6.46 (d, *J* = 8.3 Hz, 1H), 5.28 (s, 1H), 3.44 (t, *J* = 7.7 Hz, 2H), 2.88 (t, *J* = 7.7 Hz, 2H).

Q (14-206571)

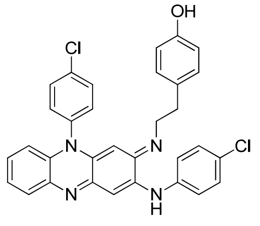

(E)-4-(2-((10-(4-chlorophenyl)-3-((4-chlorophenyl)amino)phenazin-2(10H)-ylidene)amino)-ethyl)phenol, **6Q** (General Procedure E): Purified using 12 - 60% EtOAc/Hexanes gradient. Yield: 12 mg (10%); MS (ESI$^+$), *m/z*: 551.1 & 553.1 (M+1); ^{1}H NMR (400 MHz, $(CD_3)_2SO$) δ 7.92 (dd, *J* = 8.7, 2.2 Hz, 2H), 7.70 (s, 1H), 7.60 – 7.53 (m, 2H), 7.53 – 7.45 (m, 2H), 7.44 – 7.39 (m, 2H), 7.30 (s, 2H), 6.87 – 6.77 (m, 3H), 6.72 – 6.67 (m, 2H), 6.55 (s, 1H), 5.28 (s, 1H), 3.32 (t, *J* = 7.9 Hz, 2H), 2.59 (t, *J* = 7.8 Hz, 2H).

R (21L-211916)

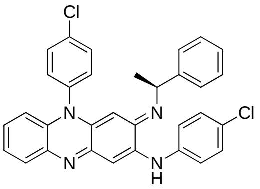

(S,E)-N,5-Bis(4-chlorophenyl)-3-((1-phenylethyl)imino)-3,5-dihydrophenazin-2-amine,[5] **6R** (General Procedure E): Yield: 15 mg (18%); MS(ESI$^+$), *m/z*: 535.0 & 537.0 (M+1); ^{1}H NMR (400 MHz, $CDCl_3$) δ 7.77 – 7.66 (m, 2H), 7.58 (d, *J* = 8.6 Hz, 1H), 7.34 – 7.27 (m, 5H), 7.23 (d, *J* = 7.3 Hz, 2H), 7.20 – 7.05 (m, 6H), 6.97 (d, *J* = 8.5 Hz, 1H), 6.85 (s, 1H), 6.47 (d, *J* = 8.2 Hz, 1H), 5.22 (s, 1H), 4.34 (q, *J* = 6.6 Hz, 1H), 1.50 (d, *J* = 6.6 Hz, 3H).

S (21R-211915)

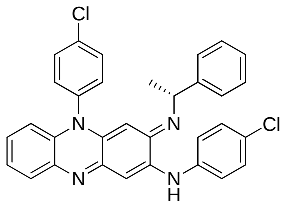

(R,E)-N,5-Bis(4-chlorophenyl)-3-((1-phenylethyl)imino)-3,5-dihydrophenazin-2-amine,[5] **6S** (General Procedure E): Yield: 15 mg (18%); MS(ESI$^+$), *m/z*: 535.0 & 537.0 (M+1); ^{1}H NMR (500 MHz, $CDCl_3$) δ 7.74 (d, *J* = 7.9 Hz, 1H), 7.71 (d, *J* = 7.9 Hz, 1H), 7.60 (d, *J* = 8.3 Hz, 1H), 7.38 – 7.29 (m, 6H), 7.26 – 7.23 (m, 2H), 7.21 – 7.17 (m, 2H), 7.16 – 7.12 (m, 1H), 7.10 – 7.06 (m, 2H), 7.01 – 6.97 (m, 1H), 6.87 (s, 1H), 6.49 (d, *J* = 8.3 Hz, 1H), 5.23 (s, 1H), 4.35 (q, *J* = 6.6 Hz, 1H), 1.51 (d, *J* = 6.5 Hz, 3H).

T (22L-212016)

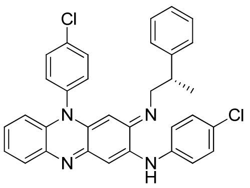

(S,E)-N,5-Bis(4-chlorophenyl)-3-((2-phenylpropyl)imino)-3,5-dihydrophenazin-2-amine, **6T** (General Procedure E): Yield: 35 mg (60%); MS (ESI$^+$), *m/z*: 549.1 & 551.1 (M+1); ^{1}H NMR (500 MHz, $CDCl_3$) δ 7.76 – 7.68 (m, 3H), 7.36 – 7.28 (m, 6H), 7.27 – 7.12 (m, 8H), 6.86 (s, 1H), 6.45 (d, *J* = 8.2 Hz, 1H), 5.25 (s, 1H), 3.33 (d, *J* = 6.4 Hz, 2H), 3.08 (h, *J* = 6.8 Hz, 1H), 1.35 (d, *J* = 6.9 Hz, 3H).

U (22R-212017)

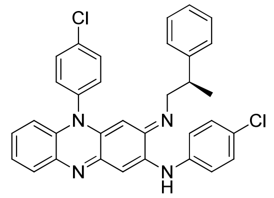

(R,E)-N,5-Bis(4-chlorophenyl)-3-((2-phenylpropyl)imino)-3,5-dihydrophenazin-2-amine, **6U** (General Procedure E): Yield: 35 mg (60%); MS (ESI$^+$), *m/z*: 549.1 & 551.1 (M+1); ^{1}H NMR (500 MHz, $CDCl_3$) δ 7.75 – 7.69 (m, 3H), 7.36 – 7.28 (m, 6H), 7.26 – 7.13 (m, 8H), 6.86 (s, 1H), 6.45 (d, *J* = 8.2 Hz, 1H), 5.25 (s, 1H), 3.32 (d, *J* = 6.8 Hz, 2H), 3.08 (h, *J* = 6.8 Hz, 1H), 1.34 (d, *J* = 6.8 Hz, 3H).

V (25L-212173)

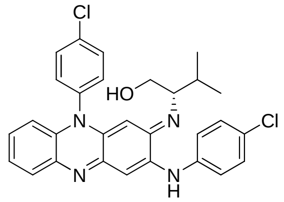

(S,E)-2-((10-(4-Chlorophenyl)-3-((4-chlorophenyl)amino)phenazin-2(10H)-ylidene)amino)-3-methylbutan-1-ol, **6V** (General Procedure E): Purified using 15 - 50% EtOAc/Hexanes gradient. Yield: 54 mg (49%); MS (ESI$^+$), *m/z*: 517.13 & 519.13 (M+1); ^{1}H NMR (400 MHz, $CDCl_3$) δ δ 7.77 – 7.65 (m, 3H), 7.42 – 7.08 (m, 10H), 6.87 (s, 1H), 6.48 (d, *J* = 7.9 Hz, 1H), 5.37 (s, 1H), 3.77 – 3.64 (m, 2H), 3.24 – 3.14 (m, 1H), 1.89 – 1.72 (m, 1H), 0.88 – 0.73 (m, 6H).

W (25R-212172)

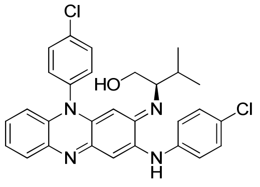

(R,E)-2-((10-(4-Chlorophenyl)-3-((4-chlorophenyl)amino)phenazin-2(10H)-ylidene)amino)-3-methylbutan-1-ol, **6W** (General Procedure E): Purified using 25 - 50% EtOAc/Hexanes gradient. Yield: 46 mg (42%); MS (ESI$^+$), *m/z*: 517.16 & 519.17 (M+1); ^{1}H NMR (400 MHz, $CDCl_3$) δ 7.74 – 7.65 (m, 3H), 7.36 – 7.12 (m, 10H), 6.87 (s, 1H), 6.48 (d, *J* = 8.1 Hz, 1H), 5.37 (s, 1H), 3.75 – 3.65 (m, 2H), 3.24 – 3.13 (m, 1H), 1.88 – 1.72 (m, 1H), 0.87 – 0.74 (m, 6H).

Table S1. Calculated physicochemical properties, predicted cell uptake and subcellular localization properties of clofazimine and related phenazine analogs with chlorophenyl substitutions.

Chemical ID	R_1	R_2	Mol. wt.	pKa	logP	Predicted ease of cell entry	Predicted intracellular localization a)
**CLOFAZIMINE**	Cl	$-CH(Me)_2$	473.4	$pK_{a1} = 9.29$<br>$pK_{a2} = 2.31$	7.30	**Poor-Moderate**<br>*Free base trapped in plasma membrane*	E G L M P
**A**	OMe	$-CH(Me)_2$	464.6	$pK_{a1} = 9.68$<br>$pK_{a2} = 2.35$	5.78	**Moderate**<br>*Free base membrane bound*	E G L M
**B**	CN	$-CH(Me)_2$	454.5	$pK_{a1} = 9.35$<br>$pK_{a2} = 2.30$	5.81	**Moderate**<br>*Free base membrane bound*	E G L M
**C**	H	$-CH(Me)_2$	404.5	$pK_{a1} = 10.06$<br>$pK_{a2} = 2.31$	6.10	**Moderate?**<br>*Free base membrane bound*	E G L M
**D**	CF_3	$-CH(Me)_2$	540.5	$pK_{a1} = 8.88$<br>$pK_{a2} = 2.32$	7.85	**Poor**<br>*Free base trapped in plasma membrane*	E G L M P
**E**	F	$-CH(Me)_2$	440.5	$pK_{a1} = 9.30$<br>$pK_{a2} = 2.31$	6.38	**Moderate**<br>*Free base membrane bound*	E G L M
**F**	COOH	$-CH(Me)_2$	492.5	$pK_{a1} = 9.77$<br>$pK_{a2} = 2.29$	5.41	**Poor**<br>*A dianionic species is membrane impermeable*	Cytosol
**G**	OH	$-CH(Me)_2$	436.5	$pK_{a1} = 9.49$<br>$pK_{a2} = 2.35$	5.49	**Moderate-good**<br>*Free base near bound/permeable boundary*	E G L M

a) E: endoplasmic reticulum, G:Golgi apparatus, L:lysosomes, M: mitochondria, P: Plasma membrane

Table S2. Observed cellular staining pattern following 72 hour incubation with clofazimine and related phenazine analogs with chlorophenyl substitutions. Scale bar is 20 µm.

ID	Notes	Transmitted microscopy images
**CLOFAZIMINE**	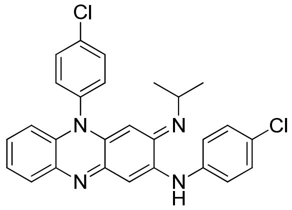<br>Orange-brown staining pattern associated with perinuclear cytoplasmic vesicles. Cells were homogeneously stained. Inferred uptake: Moderate.	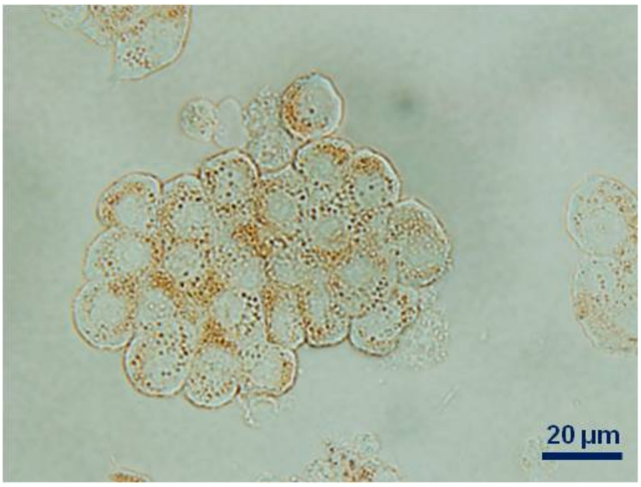
**A**	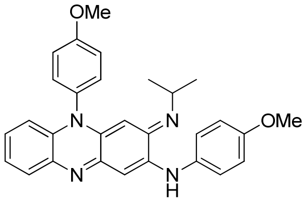<br>Compound did not exhibit any intracellular staining pattern.<br>Inferred uptake: Slow.	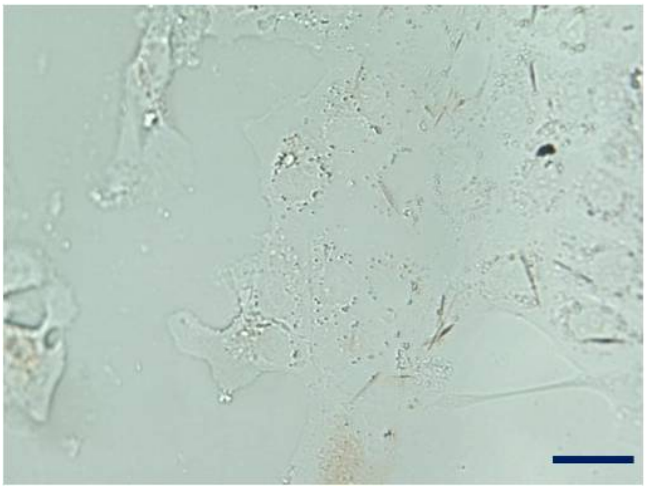
**B**	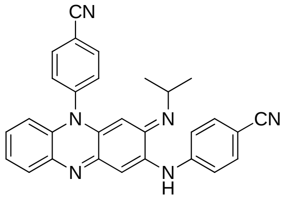<br>Compound formed large extracellular crystals. No intracellular staining pattern.<br>Inferred uptake: Very slow.	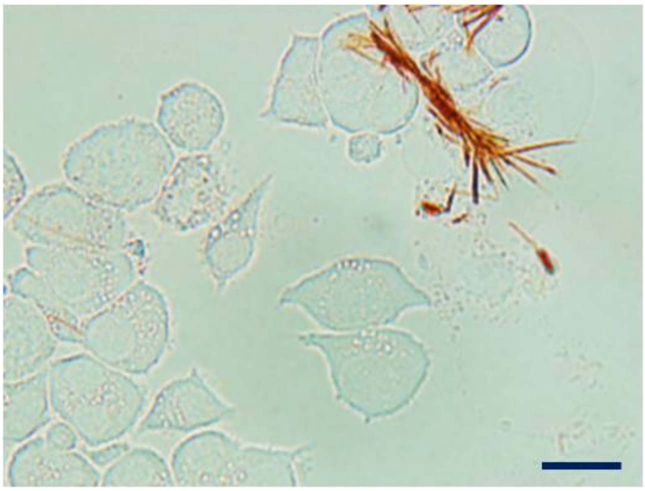

C

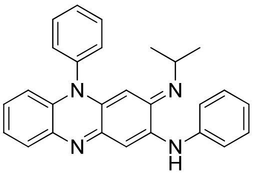

Very faint, brown perinuclear staining pattern observed across all cells.
Inferred uptake: Slow.

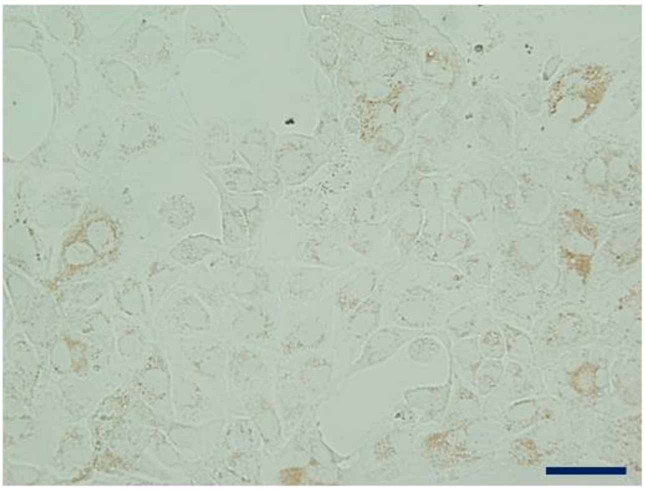

D

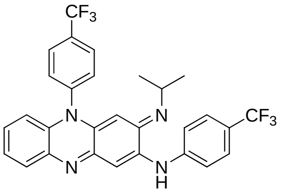

No intracellular staining pattern observed. Small percentage of cells with orange crystals.
Inferred uptake: Very slow.

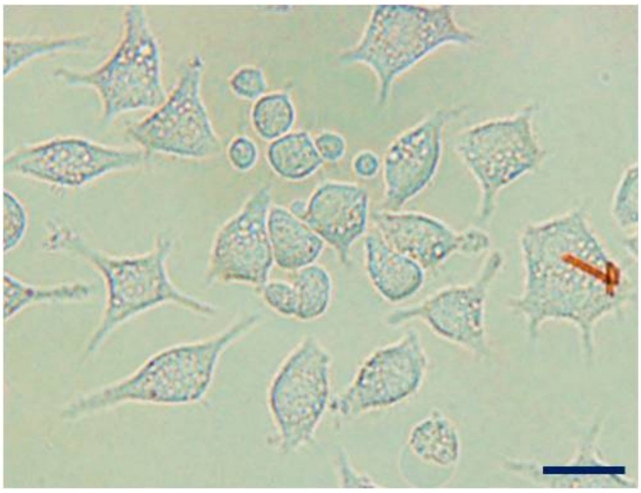

E

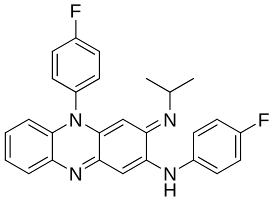

Very faint, brown perinuclear staining pattern observed across all cells.
Inferred uptake: Slow.

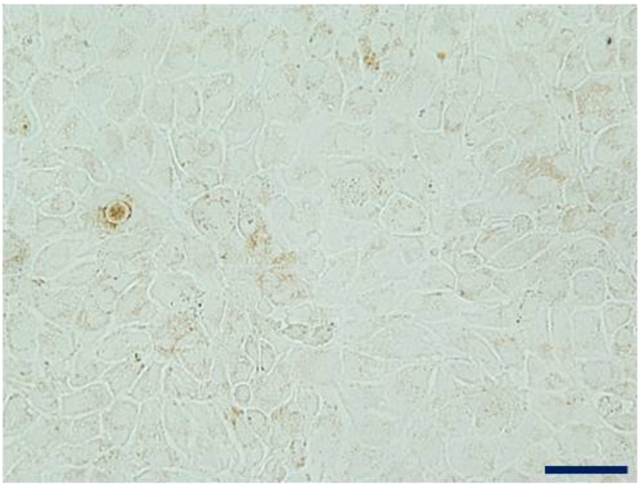

**F**	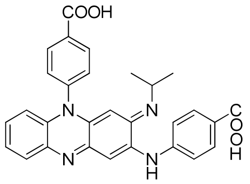 Most cells did not exhibit any staining. A few cells with associated black precipitates, likely derived extracellularly. Inferred uptake: Very slow.	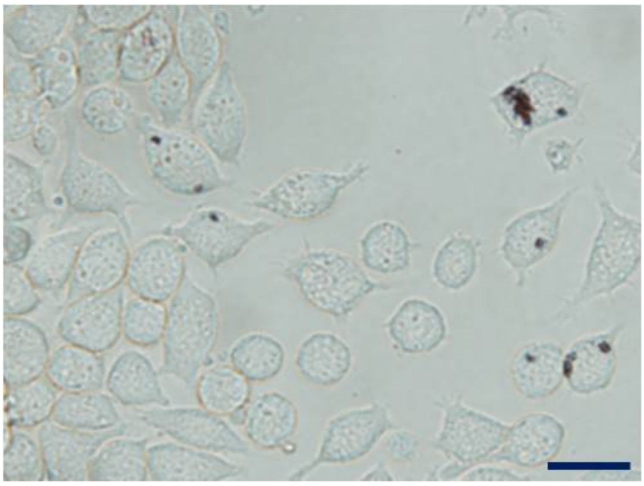
**G**	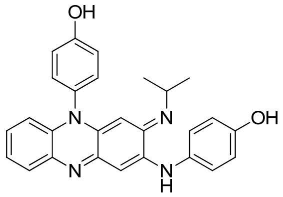 Dark brown perinuclear staining pattern associated with cytoplasmic vesicles. Inferred uptake: Good.	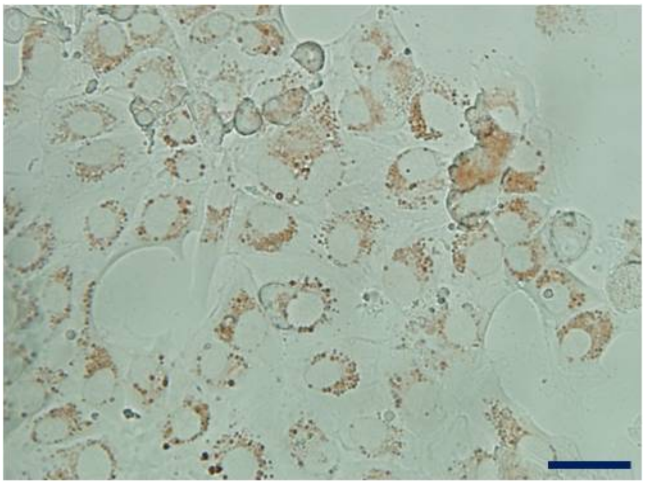

Table S3. Calculated physicochemical properties, predicted cell uptake and subcellular localization properties of clofazimine and related phenazine analogs with different R-imino substitutions.

Chemical ID	R_1	R_2	Mol. wt.	pKa	logP	Predicted ease of cell entry	Predicted intracellular localization [a)]
**H**	Cl	2-methoxyethyl	489.4	$pK_{a1} = 8.70$<br>$pK_{a2} = 2.31$	6.48	**Moderate**<br>*Free base membrane bound*	E G L M
**I**	Cl	2-hydroxyethyl	475.4	$pK_{a1} = 8.65$<br>$pK_{a2} = 2.31$	5.84	**Moderate**<br>*Free base membrane bound*	E G L M
**J**	Cl	Methyl	445.3	$pK_{a1} = 9.88$<br>$pK_{a2} = 2.33$	6.53	**Moderate**<br>*Free base membrane bound*	E G L M
**K**	Cl	n-butyl	487.4	$pK_{a1} = 9.59$<br>$pK_{a2} = 2.32$	7.85	**Poor-Moderate**<br>*Free base trapped in plasma membrane*	E G L M P
**L**	Cl	3-amino-3-oxopropyl	502.4	$pK_{a1} = 9.20$<br>$pK_{a2} = 2.32$	5.44	**Moderate**<br>*Free base membrane bound*	E G L M
**M**	Cl	2-(5-methoxy-1H-indol-3-yl)ethyl	604.5	$pK_{a1} = 9.51$<br>$pK_{a2} = 2.33$	8.49	**Poor-**<br>*Free base trapped in plasma membrane*	E G L M P
**N**	Cl	2-morpholinoethyl	544.7	$pK_{a1} = 9.16$<br>$pK_{a2} = 4.94$<br>$pK_{a3} = 2.32$	6.33	**Moderate**<br>*Free base Membrane bound*	E G L M
**O**	Cl	2-(3,4-dihydroxyphenyl)ethyl	567.5	$pK_{a1} = 9.00$<br>$pK_{a2} = 2.33$	7.94	**Poor-**<br>*Free base trapped in plasma membrane*	E G L M P
**P**	Cl	2-phenylethyl	535.5	$pK_{a1} = 9.51$<br>$pK_{a2} = 2.32$	8.54	**Poor**<br>*Free base trapped in plasma membrane*	G L P

**Q**	Cl	2-(4-hydroxyphenyl)ethyl	551.5	pK_{a1} = 9.32<br>pK_{a2} = 2.33	8.24	**Poor**<br>*Free base trapped in plasma membrane*	G L P

[a] E: endoplasmic reticulum, G:Golgi apparatus, L:lysosomes, M: mitochondria, P: Plasma membrane

Table S4. Observed cellular staining pattern following 72 hour incubation with clofazimine and related phenazine analogs possessing R-imino group substitutions. Scale bar is 20 µm.

ID	Notes	Live Cells	Fixed Cells
**H**	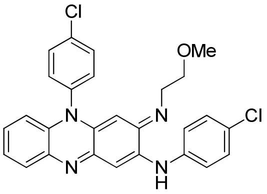<br>Brown staining associated with vesicles/membranes. Fixed cells also exhibited staining, suggesting nonspecific partitioning. Inferred uptake: Moderate	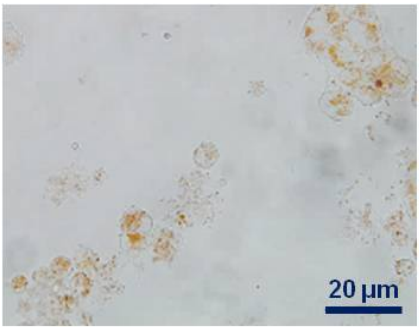	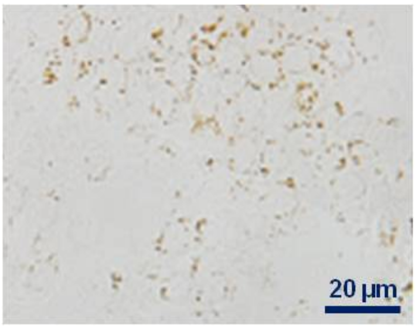
**I**	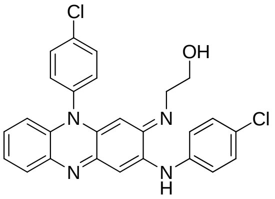<br>Dark brown, crystal-like inclusions present in live cells but absent in fixed cells. Inferred uptake: Excellent.	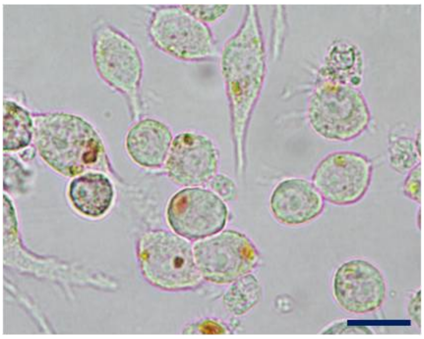	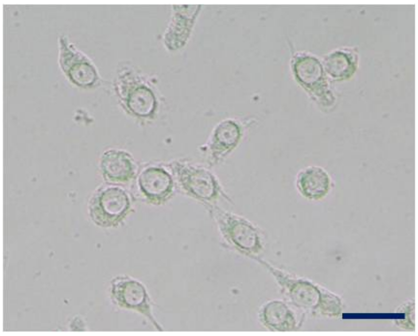
**J**	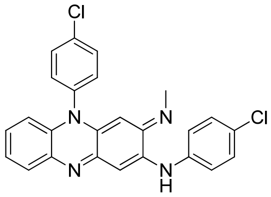<br>Orange perinuclear staining pattern associated with cytoplasmic membranes. Staining not observed in fixed cells. Inferred uptake: Good.	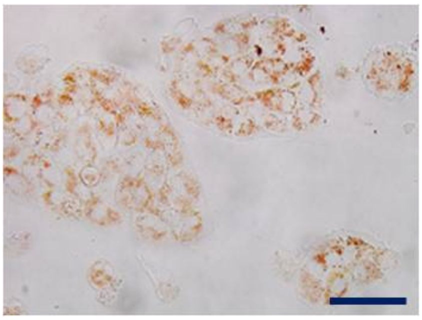	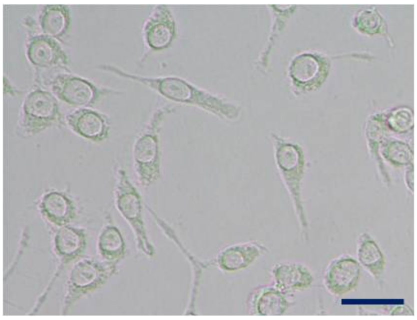

K

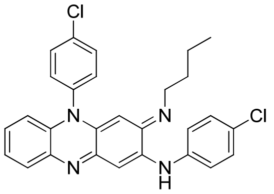

Very faint, brown perinuclear staining pattern. Inferred uptake: Slow.

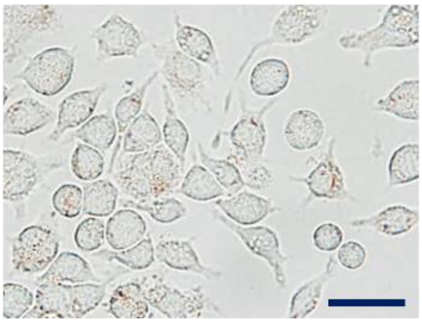

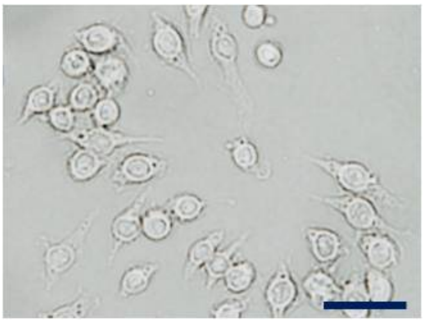

L

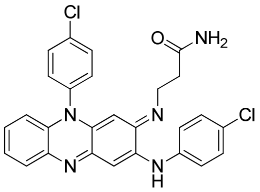

Small brown inclusions present in live cells but absent in fixed cells. Inferred uptake: Good.

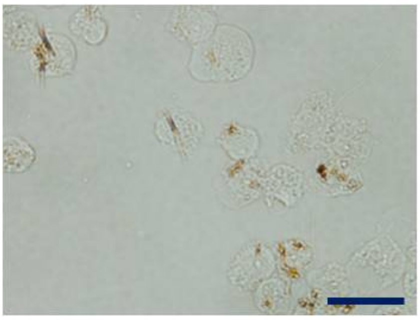

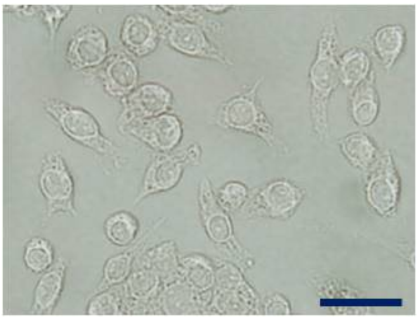

M

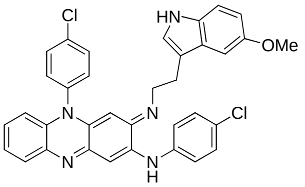

Orange-brown or purple inclusions present in live cells. Staining not observed in fixed cells. Inferred uptake: Good.

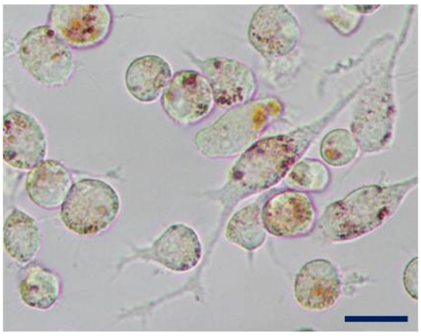

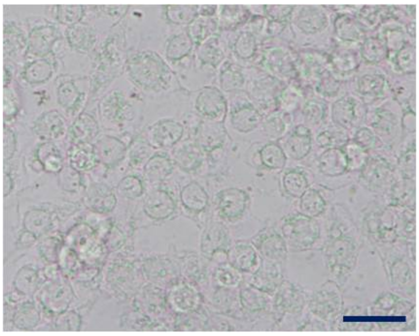

N

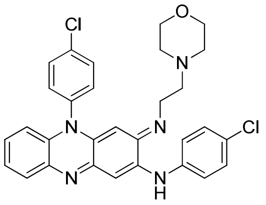

Faint orange staining pattern associated with perinuclear cytoplasmic vesicles. Cells were homogeneously stained. Staining not observed in fixed cells. Inferred uptake: Moderate.

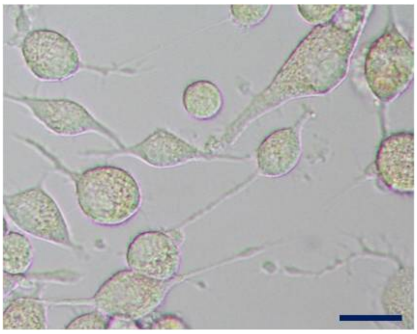

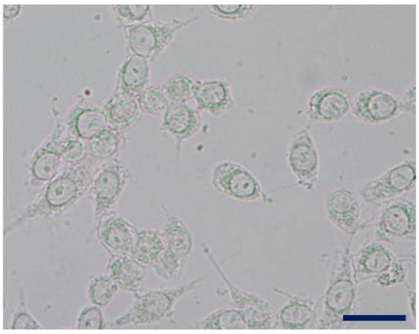

O

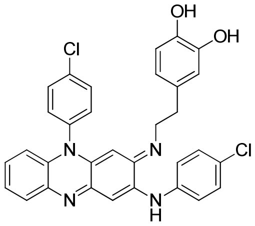

Most of cells have shown faint orange colored vesicles. Some cells contained orange-brown perinuclear staining patterns. Staining not observed in fixed cells. Inferred uptake: Good.

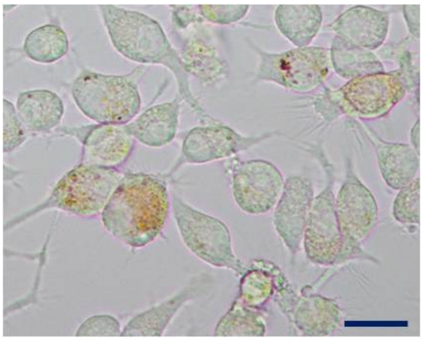

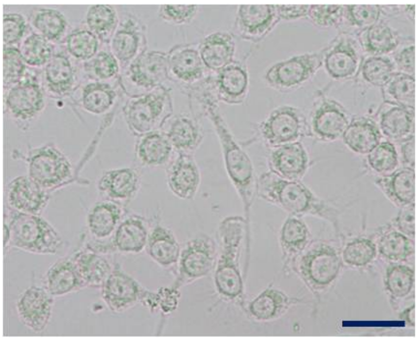

P

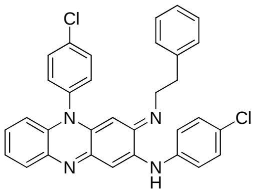

Large fibrous/crystal-like brown inclusions present in live cells but absent in fixed cells. Inferred uptake: Excellent.

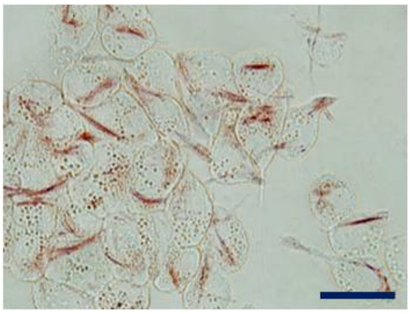

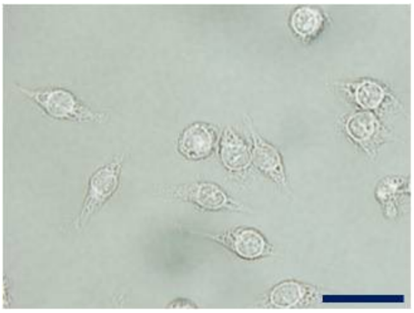

Q

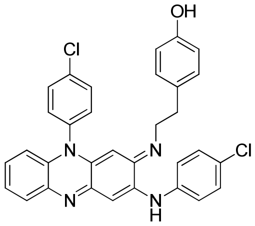

Purple-black dense cytoplasmic inclusions in live cells but absent in fixed cells. Inferred uptake: Excellent.

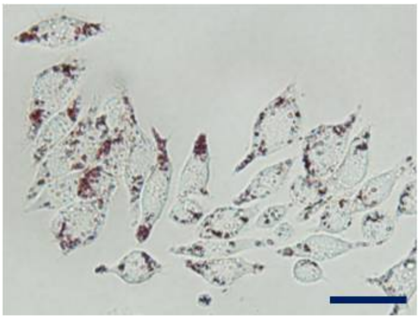

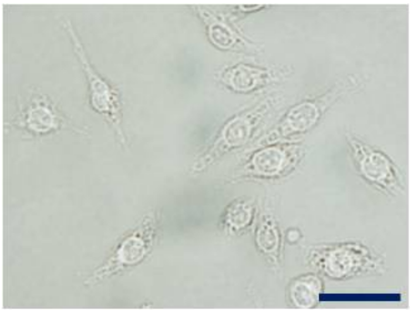

Table S5. Calculated physicochemical properties, predicted cell uptake and subcellular localization properties of clofazimine and related phenazine analogs with chiral R-imino group substitutions.

Chemical ID	**R_1**	**R_2**	Mol. wt.	pKa	logP	Predicted ease of cell entry	Predicted intracellular localization a)
**R**	Cl	(S)-1-phenylethyl	535.5	pK_{a1} = 8.35 pK_{a2} = 2.30	8.67	**Poor-** *Free base trapped in plasma membrane*	E G L M P
**S**	Cl	(R)-1-phenylethyl	535.5	pK_{a1} = 8.35 pK_{a2} = 2.30	8.67	**Poor-** *Free base trapped in plasma membrane*	E G L M P
**T**	Cl	(S)-2-phenylpropyl	549.5	pK_{a1} = 9.46 pK_{a2} = 2.32	8.91	**Poor-** *Free base trapped in plasma membrane*	E G L M P
**U**	Cl	(R)-2-phenylpropyl	549.5	pK_{a1} = 9.46 pK_{a2} = 2.32	8.91	**Poor-** *Free base trapped in plasma membrane*	E G L M P
**V**	Cl	(S)-1-hydroxy-3-methylbut-2-yl	517.5	pK_{a1} = 8.28 pK_{a2} = 2.30	7.14	**Poor-Moderate** *Free base trapped in plasma membrane*	E G L M P
**W**	Cl	(R)-1-hydroxy-3-methylbut-2-yl	517.5	pK_{a1} = 8.28 pK_{a2} = 2.30	7.14	**Poor-Moderate** *Free base trapped in plasma membrane*	E G L M P

a) E: endoplasmic reticulum, G:Golgi apparatus, L:lysosomes, M: mitochondria, P: Plasma membrane

Table S6. Observed cellular staining pattern following 72 hour incubation with clofazimine and related phenazine analogs possessing chiral R-imino group substitutions. Scale bar is 20 µm.

ID	Notes	Live Cells	Fixed Cells
**R**	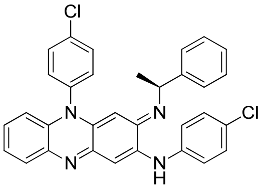 Orange-brown staining pattern associated with perinuclear cytoplasmic vesicles in live cells. No staining found in Fixed cells. Inferred uptake: Good.	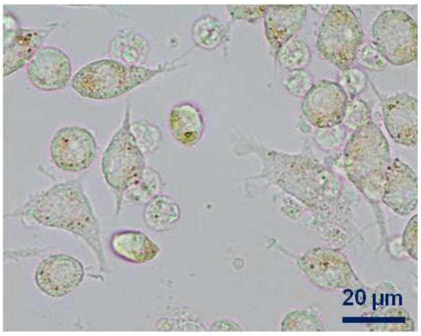	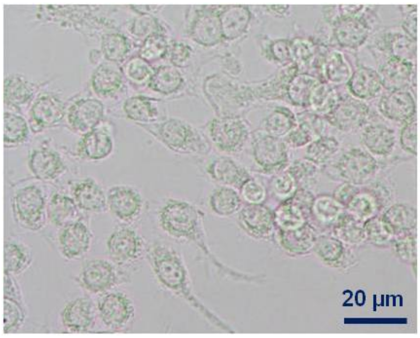
**S**	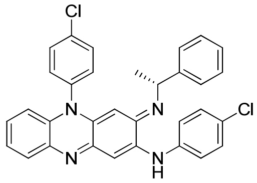 Orange-brown staining pattern associated with perinuclear cytoplasmic vesicles in live cells. Fixed cells do not show any staining. Inferred uptake: Good.	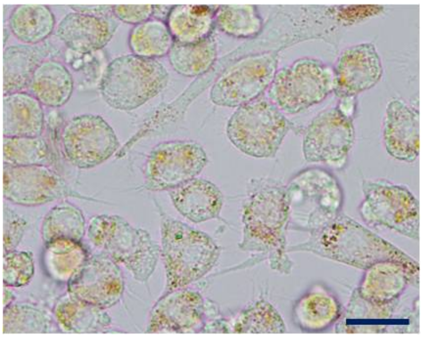	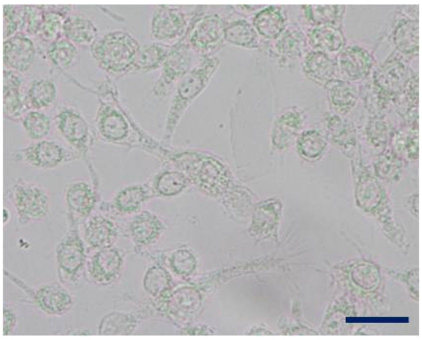
**T**	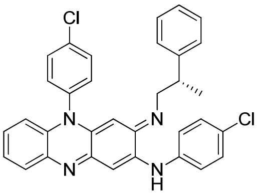 Faint orange perinuclear vesicles are present in live cells. Staining not observed in fixed cells. Inferred uptake: Moderate.	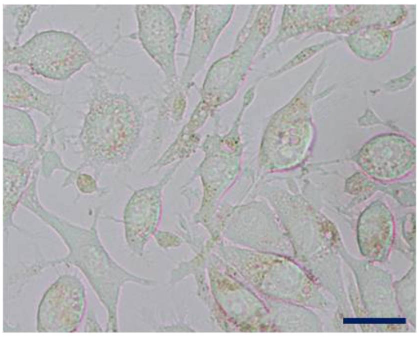	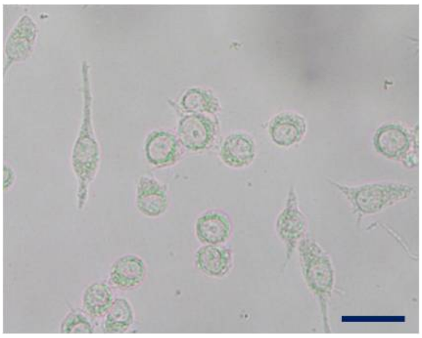

**U**	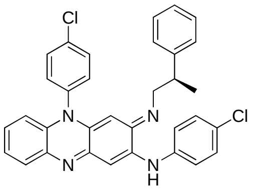 Fibrous/crystal-like brown inclusions with orange perinuclear vesicles are present in live cells but absent in fixed cells. Inferred uptake: Good.	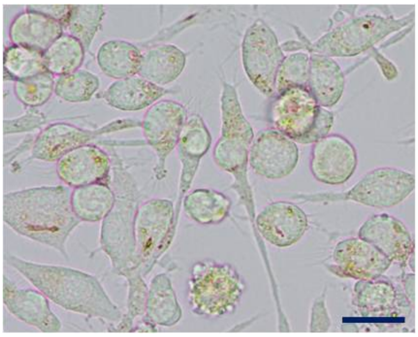	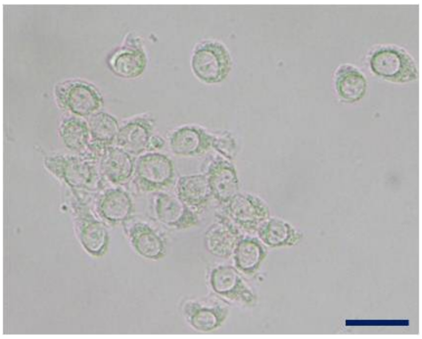
**V**	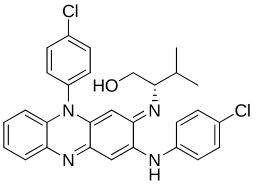 Brown crystal-like inclusions appeared in live cells. Staining not observed in fixed cells. Inferred uptake: Good.	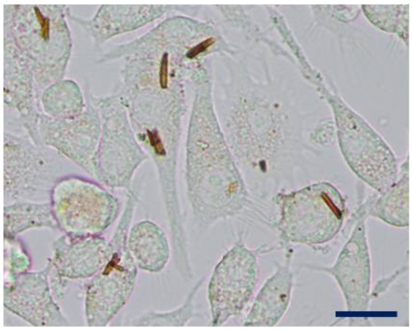	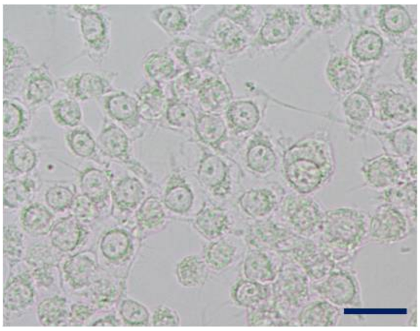
**W**	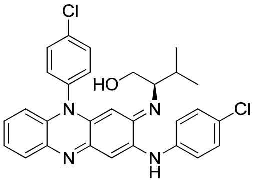 Brown crystal-like inclusions are shown in live cells but absent in fixed cells. Inferred uptake: Good.	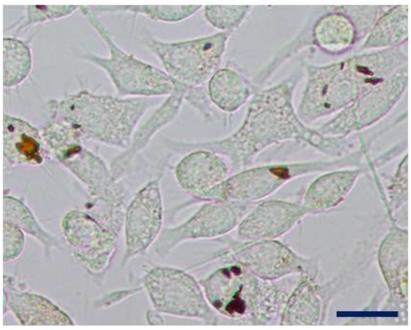	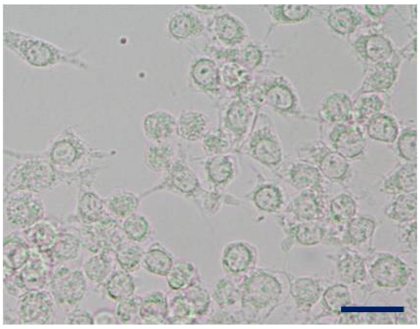

References

[1] a)V. C. Barry, M. L. Conalty, *Am. Rev. Tuberc.* **1958**, *78*, 62-73; b)J. Belton, M. Conalty, C. O'Callaghan, J. O'Sullivan, D. Twomey, in *Proceedings of the Royal Irish Academy. Section B: Biological, Geological, and Chemical Science*, JSTOR, Ireland, **1961**, pp. 15-22.
[2] J. áO'Sullivan, *J. Chem. Soc.* **1958**, 859-863.
[3] J. F. O'Sullivan, M. L. Conalty, N. E. Morrison, *J. Med. Chem.* **1988**, *31*, 567-572.
[4] A. C.-G. G.-A. JORI, H. ZEVIO, *Farmaco. Edizione scientifica.* **1962**, *17*, 308-319.
[5] J. F. O'Sullivan, *J. Chem. Res., Miniprint,* **1984**, *52.*
[6] C. Hansch, A. Leo, *Wiley, New York,* **1979**, pp. 18-43.
[7] a)R. W. Horobin, F. Rashid-Doubell, J. D. Pediani, G. Milligan, *Biotech. Histochem.* **2013**, *88*, 440-460; b)R. W. Horobin, F. Rashid-Doubell, *Biotech. Histochem.* **2013**, *88*, 461-476.
[8] J. Baik, G. R. Rosania, *Mol. Pharm.* **2011**, *8*, 1742-1749.
[9] a)R. Oldenbourg, *Nature* **1996**, *381*, 811-812; b)R. Oldenbourg, G. Mei, *J. Microsc.* **1995**, *180*, 140-147; c)M. Shribak, R. Oldenbourg, *Appl. Opt.* **2003**, *42*, 3009-3017.
[10] a)S. B. Mehta, M. Shribak, R. Oldenbourg, *J. Opt.* **2013**, *15*, 094007-094020; b)B. S. DeMay, N. Noda, A. S. Gladfelter, R. Oldenbourg, *Biophys. J.* **2011**, *101*, 985-994.
[11] H. Abdi, L. J. Williams, *Wiley Interdisciplinary Reviews: Computational Statistics.* **2010**, *2*, 433-459.
[12] a)Z.-B. Xu, Y. Lu, Z.-R. Guo, *Synlett.* **2003**, *4*, 564-566; b)P. Kirsch, A. Schonleben-Janas, R. H. Schirmer, *Liebigs Annalen* **1995**, *1995*, 1275-1281.
